# Schwann cells support oncogenic potential of pancreatic cancer cells through TGFβ signaling

**DOI:** 10.1038/s41419-019-2116-x

**Published:** 2019-11-25

**Authors:** Elodie Roger, Sylvie Martel, Adrien Bertrand-Chapel, Arnaud Depollier, Nicolas Chuvin, Roxane M. Pommier, Karam Yacoub, Cassandre Caligaris, Victoire Cardot-Ruffino, Véronique Chauvet, Sophie Aires, Kayvan Mohkam, Jean-Yves Mabrut, Mustapha Adham, Tanguy Fenouil, Valérie Hervieu, Laura Broutier, Marie Castets, Cindy Neuzillet, Philippe A. Cassier, Richard Tomasini, Stéphanie Sentis, Laurent Bartholin

**Affiliations:** 1 Université de Lyon, Université Claude Bernard Lyon 1, INSERM 1052, CNRS 5286, Centre Léon Bérard, Centre de recherche en cancérologie de Lyon (CRCL), Lyon, 69373 France; 2 Hospices Civils de Lyon, Croix Rousse hospital, Claude-Bernard Lyon 1 University, Department of General Surgery & Liver Transplantation, Lyon, France; 3 Hospices Civils de Lyon, Edouard Herriot hospital, Claude-Bernard Lyon 1 University, Department of General Surgery & Liver Transplantation, Lyon, France; 4 Hospices Civils de Lyon Institute of Pathology EST, CRCL INSERM U1052, University Lyon 1, Lyon, France; 5 Medical Oncology Department, Curie Institute, Versailles Saint-Quentin University, 35 rue Dailly, 92210 Saint Cloud, France; 6 Departement d’Oncologie Médicale, Centre Léon Bérard, Lyon, 69008 France; 7 Aix-Marseille Université, Institut Paoli-Calmettes, INSERM U1068, CNRS UMR 7258, Centre de Recherche en Cancérologie de Marseille, Marseille, France; 8 Present Address: Clinical Research Division, Fred Hutchinson Cancer Research Center, Seattle, WA USA

**Keywords:** Cancer microenvironment, Oncogenes

## Abstract

Pancreatic ductal adenocarcinoma (PDAC) is one of the solid tumors with the poorest prognosis. The stroma of this tumor is abundant and composed of extracellular matrix and stromal cells (including cancer-associated fibroblasts and immune cells). Nerve fibers invading this stroma represent a hallmark of PDAC, involved in neural remodeling, which participates in neuropathic pain, cancer cell dissemination and tumor relapse after surgery. Pancreatic cancer-associated neural remodeling is regulated through functional interplays mediated by physical and molecular interactions between cancer cells, nerve cells and surrounding Schwann cells, and other stromal cells. In the present study, we show that Schwann cells (glial cells supporting peripheral neurons) can enhance aggressiveness (migration, invasion, tumorigenicity) of pancreatic cancer cells in a transforming growth factor beta (TGFβ)-dependent manner. Indeed, we reveal that conditioned medium from Schwann cells contains high amounts of TGFβ able to activate the TGFβ-SMAD signaling pathway in cancer cells. We also observed in human PDAC samples that high levels of TGFβ signaling activation were positively correlated with perineural invasion. Secretome analyses by mass spectrometry of Schwann cells and pancreatic cancer cells cultured alone or in combination highlighted the central role of TGFβ in neuro-epithelial interactions, as illustrated by proteomic signatures related to cell adhesion and motility. Altogether, these results demonstrate that Schwann cells are a meaningful source of TGFβ in PDAC, which plays a crucial role in the acquisition of aggressive properties by pancreatic cancer cells.

## Introduction

Pancreatic ductal adenocarcinoma (PDAC) is one of the most aggressive malignant diseases with a 5-year median overall survival rate lower than 8%^1^. By 2030, PDAC will be the second leading cause of cancer-related deaths in Western countries^2,3^. Late diagnosis, high metastatic potential of PDAC cells and low efficacy of currently available therapies are responsible for this poor prognosis^4^.

PDAC is characterized by an abundant stroma that can represent over 80% of the tumor volume. This desmoplastic stroma is composed of extracellular matrix (ECM) components (such as collagens, hyaluronic acid, fibronectin) and various cell types including cancer-associated fibroblasts (CAFs)^5^ and immunosuppressive immune cells (regulatory T cells, M2 macrophages, myeloid-derived suppressor cells)^6^. More surprisingly, many nerve fibers are systematically observed by pathologists within the PDAC tumor, whereas nerve fibers in normal pancreas are confined to fat pads surrounding the organ. This observation is part of a more general concept termed pancreatic cancer-associated neural remodeling (PANR)^7–10^. This PDAC hallmark is closely related to multiple clinicopathological features^11^ and probably linked to genomic alterations in axon guidance signaling genes that have been reported in PDAC^12^. PANR consists on the one hand in a phenomenon called neural remodeling (NR) characterized by an increase in the size and density of nerve fibers within tumors compared to nerves present around a healthy pancreas, as well as changes in signal transmission abilities^13^. NR is thought to be responsible for the intense neuropathic pain that is frequently experienced by patients with PDAC^14^. On the other hand, tumor cells infiltrating nerve fibers are detectable in 95% of PDAC patients. This phenomenon called perineural invasion (PNI) is correlated with pain, local recurrence and distant metastasis in PDAC^14^.

The molecular dialog between cancer cells, nerves and surrounding Schwann cells, and other stromal cells remains scarcely documented. However, it has been shown that (i) cancer cells secrete chemo attracting factors for Schwann cells, such as NGF and ARTN^15^, CXCL12/SDF-1^16^, and IL6^17^, (ii) stromal cells such as CAFs and immune cells then produce secreted factors such as SLIT2^18^ or LIF^19^, which stimulate proliferation and migration of Schwann cells, and finally (iii) Schwann cells reciprocally induce tumor cell migration through the secretion of GDNF^20^, as well as via the NCAM1-mediated cell–cell interaction^21^. Functionally, it was shown in a genetic PDAC model that sensory neuron ablation results in a slower cancer initiation and progression associated with decreased PANR, highlighting that nerve fibers actively support tumor growth^22^.

Among the other secreted factors potentially involved in neural remodeling, the transforming growth factor beta (TGFβ) represents an attractive candidate^23,24^. TGFβ was shown to stimulate the expression of some factors involved in neural development, homeostasis or remodeling such as NGF^25^, ARTN^15,26^, CX3CR1^27–29^, and ROBO1^30^. Interestingly, it has recently been shown that a low ROBO2/ROBO1 ratio in pancreatic cancer cells is correlated with TGFβ signaling activation in myofibroblasts and associated with a poorer prognosis^31^. TGFβ is also described for its neuroprotective activities^32–35^, and as being involved in the perception of pain^36–38^.

Hence, it is tempting to speculate that TGFβ may play a direct role in PANR. To that end, we herein explored the potential role of TGFβ as a direct modulator of cancer-nerve cell interactions, using co-cultures and conditioned media from PDAC (Capan-2) and Schwann (sNF96.2) cell lines in migration and invasion assays. Owing to the abundance of secreted factors within the PDAC desmoplastic stroma, we also characterized in a large-scale screening (mass spectrometry) the secretomes of the Schwann cells cultured alone or in combination with pancreatic cancer cells.

## Material and methods

### Cell culture

The human pancreatic cancer cell line, Capan-2, was obtained from the American Type Culture Collection (ATCC), and Schwann cells (sNF96.2) were kindly provided by Dr. Richard Tomasini (Centre de Recherche en Cancérologie de Marseille, Marseille, France). sNF96.2 Schwann cells were derived from neurofibromatosis type-1 disease. Capan-2 and sNF96.2 cell lines were cultured in DMEM supplemented with 10% fetal bovine serum (FBS) (Gibco) and 1% of penicillin/streptomycin (Gibco). These two cell lines had recently been tested negative for mycoplasma.

Human Schwann cells (HSwC) were obtained from iXcells Biotechnologies (Cat#10HU-188), isolated from human spinal nerve and cryopreserved at P1. HSwC were cultured in Schwann Cell Basal Medium (Cat#MD-0055B), supplemented with 10% fetal bovine serum (FBS,Cat#MD-0094), Schwann Cell Growth Supplement (SCGS,Cat#MD-0055S), and 1% antibiotic-antimytotic (Cat#MD-0095).

The human hTERT NF1 ipn02.3 2λ cell line^39^ was obtained from the ATCC (Cat#CRL-3392) from a healthy sural nerve (sciatic nerve) in a case of plexiform neurofibroma. ipn02.3 2λ cells were cultured in DMEM supplemented with 10% fetal bovine serum (FBS) (Gibco), 2 mM L-glutamine(Gibco) and 1% penicillin/streptomycin (Gibco).

### Conditioned medium production

Conditioned medium (CM) was collected from cell cultures of Capan-2 cells (Capan-2 CM) and sNF96.2 cells (sNF96.2 CM) cultured alone or in combination (Capan-2/sNF96.2 CM). For CM production of Capan-2 and sNF96.2 cells cultured alone, 300,000 cells of each cellular type were seeded onto a six-well tissue culture plate in complete DMEM. Following overnight culture for cell attachment, the medium was replaced by DMEM containing 0.5% FBS. In all experiments, except for Fig. S5 (in which conditioned medium was harvested at 72 h), conditioned media were collected after 24 h of culture, centrifuged at 1,200 rpm and cell supernatants were harvested and stored at −80 °C.

For CM production from Capan-2 cells co-cultured with sNF96.2 cells, 300,000 cells of each cell line were seeded into co-culture plates, pores size 0.4 μm (300,000 Capan-2 cancer cells into the lower compartment and 300,000 sNF96.2 cells into the upper compartment) and processed in the same conditions as conditioned medium produced with Capan-2 and sNF96.2 cells cultured alone.

### 3D migration assay

The 3D migration assay was performed with Capan-2 cells and sNF96.2 cells. 2,000,000 cells/mL of each cell line were resuspended in cold ECM gel (Sigma-Aldrich), and drops of 20,000 cells/10 μl were placed in a 12-well tissue culture plate at a 1-mm distance from each other and connected via an ECM bridge. An empty ECM gel drop in front of the ECM drop containing Capan-2 cells was used as a negative control. After 15 min of ECM gel hardening at 37 °C, DMEM medium supplemented with 1% FBS was added in each well, and cells were treated or not with 10 μM SB-431542 TβRI inhibitor. The migration of Capan-2 cells onto the ECM bridge toward sNF96.2 cells was monitored for 15 days. The 3D cultures were monitored using an inverted microscope (Zeiss Axiovert 200 M) at different timepoints (0, 5, 7, and 15 days). These experiments were repeated three times.

### Cell migration and invasion assays

Cell migration and invasion assays were performed using 24-well transwell chambers (Boyden chambers, Corning). These experiments were performed using co-cultures of Capan-2 cells and sNF96.2 cells or using CM from these cell lines. For co-culture experiments, 50,000 Capan-2 cells were seeded into the upper chamber of an 8-μm pore size insert in the 24-well plate, with (invasion assay) or without (migration assay) ECM gel (cells were resuspended in matrigel, diluted in DMEM FBS-free at a concentration of 5 mg/mL; Sigma-Aldrich) and 100,000 sNF96.2 cells or 100,000 Capan-2 cancer cells (used as a control condition) were seeded into the lower compartment. For CM experiments, sNF96.2 cells were replaced by sNF96.2 CM or by Capan-2 CM (used as a control condition) in the lower compartment.

Capan-2 cells were treated or not with 10 ng/mL TGFβ and 10 μM SB-431542 TβRI inhibitor in indicated wells. In addition, each well was treated with 8 μM of 5-fluorouracil to avoid cell proliferation and 5% FBS chemoattractant was placed in the well below. Capan-2 cells were left to migrate or invade the bottom chamber containing cells for 72 h. Wells were fixed with cold methanol and stained with a 0.1% crystal violet solution. The cells on the surface of the upper membrane which had not migrated were removed with a cotton tip. Each well was photographed and subsequently analyzed using the ImageJ software (area calculation of migratory and invasive Capan-2 cells attached to the lower membrane surface). Migration experiments were repeated at least four times and invasion experiments were repeated at least three times.

### Cell proliferation assay

Cell proliferation was determined using an IncuCyte® ZOOM Imaging System (Essen Bioscience). Capan-2 cells were transduced using the Nuclight Lentivirus Reagent, according to the manufacturer’s instructions, and providing homogeneous expression of a nuclear-restricted mKate protein (far-red fluorescent protein). The Capan-2-nRFP^+^ stable cell line was then established by 1 μg/mL puromycin selection.

For proliferation assays, 50,000 Capan-2-nRFP^+^ cells were seeded onto 48-well culture plates in complete DMEM. Following their overnight culture for cell attachment, serum deprivation was performed for 1 h. Serum-free medium was replaced by CM from sNF96.2 cells, CM from Capan-2 cancer cells. Capan-2-nRFP^+^ cells were automatically counted during 72 h. For the quantification of the relative rate of cell proliferation, the number of Capan-2-nRFP^+^ cells was normalized against the T0 Capan-2-nRFP^+^ cell count of the respective well. These experiments were performed in triplicate and repeated three times.

### Wound healing assay

For wound healing assays, 120,000 Capan-2 cells were seeded onto 48-well tissue culture plates in complete medium. Following their overnight culture for cell attachment, serum deprivation was performed for 1 h and cells were pre-treated with 10 μM SB-431542 TβRI inhibitor in the indicated wells for 1 h. Capan-2 cells were then wounded using a 200 μl pipette tip and washed with serum-free DMEM. Gently, sNF96.2 CM, Capan-2 CM, or FBS 0.5% DMEM (used as internal control) were added, and Capan-2 cells were treated or not with 10 ng/mL TGFβ and 10 μM SB-431542 TβRI inhibitor. In addition, each well was treated with 8 μM of 5-fluorouracil to avoid cell proliferation. Using an IncuCyte® ZOOM Imaging System (Essen Bioscience), cells were monitored for at least 24 h. For quantification of wound areas, three fields per well/per experiment, were visualized at different timepoints (0 and 12 h) using the ImageJ software. Percentages of wound closure were determined using the following equation: % of wound closure = ((wound area at time X−initial wound area) × 100)/initial wound area. These experiments were performed in triplicate and repeated at least three times.

### Immunofluorescence

Cells were treated and grown on 48-well plates (conditioned medium experiments) or on glass coverslips in the bottom compartment of 6-well co-culture plates (co-cultures experiments, pore size 0.4 μm) and fixed with 4% paraformaldehyde. Cells were then permeabilized with 0.1% Triton X-100 in PBS for 20 min and blocked with 5% FBS in PBS for 30 min at room temperature (RT). The primary antibodies used were: mouse monoclonal antibodies against β-CATENIN (1:100; BD Biosciences #610153), E-CADHERIN (1:300; BD Biosciences #610181) and rabbit monoclonal antibody SMAD2 (1:100; Cell signaling D43B4). Secondary labeled antibodies were goat antibody against mouse IgG, Alexa Fluor 488 dye-conjugated (1:500; Invitrogen) and goat antibody against rabbit IgG, Alexa Fluor 594 dye-conjugated (1:500; Invitrogen). F-ACTIN was stained with phalloidin-TRITC (1:500; Sigma-Aldrich). DNA was stained with DAPI (1 μg/mL; Sigma-Aldrich). Fluorescent images were taken with the Zeiss Axio Imager M2 upright microscope (for co-culture experiments) or with the Axio Observer inverted microscope (for conditioned medium experiments). Images were processed using Zeiss Zen and ImageJ software.

### ELISA

Protein levels of TGFβ1 were measured in CM from Capan-2 and sNF96.2 cells using the human TGFβ1 Quantikine ELISA Kit (R&D Systems) according to the manufacturer’s instructions.

### Protein extraction and Western blotting

Total protein extracts (30 μg) were prepared using RIPA lysis buffer (50 mM Tris, pH 7.5, 150 mM NaCl, 1% Nonidet P-40, 0.5% sodium deoxycholate, 0.1% SDS and commercial protease and phosphatase inhibitor cocktail tablets (Roche)) and were subjected to electrophoresis on SDS–PAGE. The separated proteins were transferred onto PVDF membranes (Millipore) by electroblotting. Western blots were visualized using the ECL Western detection system (GE Healthcare). Images were acquired using a ChemiDoc gel imager (BioRad, Universal Hood III). The primary and secondary antibodies used were:

**Table 1:** 

P-SMAD2,	Rabbit monoclonal, 1:1000	(Cell Signalling, 138D4)
SMAD2,	Rabbit monoclonal, 1:1000	(Cell Signalling, D43B4))
GAPDH,	Mouse monoclonal, 1:20 000	(Abcam, #8245)
Rabbit IgG secondary antibody,	Goat, 1:10 000	(Immuno Reagents Inc.)
Mouse IgG secondary antibody,	Goat, 1:10 000	(DakoCytomation)

### Proteomic analyses - LFQ mass spectrometry

#### Sample preparation for LFQ mass spectrometry

For mass spectrometry experiments, CM from Capan-2 and sNF96.2 cells, cultured alone or in combination (see Conditioned medium production section), were collected after 24 h of growth in FBS-free DMEM. Three 12 mL CM aliquots were used for each condition, centrifuged at 1 200 rpm for 3 min (for cell debris removal), and concentrated using Amicon Ultra-4 centrifugal filter devices (3,000 MWCO cutoff) (Millipore) by successive spinning (4 mL of CM per spinning) at 5,976 rpm for 25 min. Protein concentrations were determined by Bradford assay and 50 μg of proteins were used for mass spectrometry sample preparation. Following the protein precipitation step (using trichlororoacetic acid 20% in volume, overnight at 4 °C), samples were washed in acetone and solubilized in 8 M urea. Samples were then reduced (Tris(2-CarboxyEthyl)Phosphine, 5 mM, 57 °C, 1 h), alkylated (iodoacetamide, 10 mM, RT, 45 min), and digested (5 h at 37 °C with LysC and overnight at 37 °C with trypsin). Peptides were next desalted using C18 spin columns (Harvard Apparatus), dried and resuspended in 100 μL H_2_O/ACN-98/2-0.1% AF. Ten microliters of each sample were used to determine peptide concentration (Pierce™ Quantitative Fluorometric Peptide Assay, Thermofisher). Five micrograms were collected, dried and diluted in 18 μl H_2_O/ACN-98/2-0.1% AF. In total 2 μL of a 2 pmol/μL Cytochrome C solution used as an internal control, were added to each sample. Hence, 250 ng of peptides and 200 fmol of Cytochrome C, per sample, were injected into the mass spectrometer (Q Exactive HF, Thermo Scientific, Protein Science Facility UMS3444, Lyon, France).

#### Mass spectrometry analysis

Samples were analyzed in a Label Free quantitation strategy using an Ultimate 3000 nano-RSLC (Thermo Scientific, San Jose California) coupled online with a Q Exactive HF mass spectrometer via a nano-electrospray ionization source (Thermo Scientific, San Jose California). The proteomics results are representative of three biological replicates per sample, subdivided into three technical replicates (*i.e*. 9 technical replicates experiment for each sample) (see Fig. S8). The samples were loaded onto a C18 Acclaim PepMap100 trap-column 75 µm ID × 2 cm, 3 µm, 100 Å, (Thermo Scientific) for 3 min at 5 µL/min with 2% ACN, 0.05% TFA in H_2_O and then separated on a C18 Acclaim Pepmap100 nano-column, 50 cm × 75 μm i.d, 2 μm, 100 Å (Thermo Scientific) with a 120 min gradient at a flow rate of 300 nL/min: 100 min from 4% to 20% buffer B (A: 0.1% FA in H_2_O, B: 0.1% FA in 100% AcN), 20 min from 20% to 32% of B and then from 32% to 95% of B in 2 min, maintained for 10 min and back to the initial conditions in 1 min for 14 min. The oven temperature was kept constant at 40 °C.

Samples were analyzed with a TOP20 HCD method. Briefly, MS data were acquired using a data-dependent strategy selecting the fragmentation events based on the 20 most abundant precursor ions in the survey scan (375-1,800 Th). The resolution of the survey scan was 60,000 at m/z 200 Th and for MS/MS scan the resolution was set to 15,000 at m/z 200 Th. The Ion Target Value for the survey scans in the Orbitrap and the MS/MS scan were set at 3E6 and 1E5 respectively and the maximum injection time was set at 60 ms for both MS and MS/MS scan. Parameters for acquiring HCD MS/MS spectra were as follows; normalized collision energy = 27 and an isolation window width of 2 m/z. The precursors with unknown charge state, charge state of 1 and 8 or greater than 8 were excluded. Peptides selected for MS/MS acquisition were then placed on an exclusion list for 30 s using the dynamic exclusion mode to limit spectrum duplication.

#### Data analysis

Protein identification was performed using the SEQUEST HT algorithm integrated in Proteome Discover 2.2 (Thermo Scientific) and MS/MS scans were examined against a human dataset (SwissProt_ juillet 2018). Precursor mass tolerance was set at 10 ppm and fragment mass tolerance was set at 0.02 Da, and up to 2 missed cleavages were allowed. Oxidation (M), acetylation (Protein N-terminus) were set as a variable modification, and Carbamidomethylation (C) as a fixed modification. False discovery rate (FDR) of peptide identification was calculated by the Percolator algorithm method, and a cut-off FDR value of 1% was used. Protein quantification was conducted by Label Free Quantification (LFQ) approach, and LFQ abundance values were obtained for each sample, normalized against the total amount of peptides and were transformed into log10 scale. Samples were grouped according to the CM type (i.e., 3 groups: Capan-2 CM, sNF96.2 CM, and Capan-2/sNF96.2 CM). Log10 LFQ abundance values were used to generate heatmaps (Morpheus, Broad Institute, USA).

Differential protein abundance was analyzed using ratio calculations (pairwise ratios were calculated as median of all possible pairwise ratios calculated between replicates of all connected peptides) and validated using an ANOVA (background based) statistical analysis.

In these settings, we performed the following ratio calculations: ratio sNF96.2/Capan-2/sNF96.2 CM; ratio sNF96.2/Capan-2 CM/Capan-2 CM; and ratio Capan-2 CM/sNF96.2 CM. Note that the value 100 is used as the maximum allowed fold change. Significant differences between conditions were defined by an adjusted *p*-value < 0.05.

Gene ontology (GO) enrichment analysis of biological processes of the significantly different proteins were performed using the PANTHER (protein annotation through evolutionary relationship) classification system (i.e. web site: http://geneontology.org). PANTHER overrepresentation tests were performed against the GO Ontology database (reference list: Homo sapiens) and significance was assessed with Fisher’s exact test (using the Bonferroni correction for multiple hypothesis testing). Protein-protein interactions were explored using STRING analysis (reference database: Homo sapiens database; i.e., web site: string-db.org), with a minimum required interaction score set at a high confidence level of 0.700. Protein clusters were defined using the MCL algorithm with an inflation parameter set at 3.

### Clonogenic survival assay

For clonogenic survival assays, 1000 Capan-2 cells were seeded onto 12-well tissue culture plates in 1 mL of complete DMEM. Following their overnight culture for cell attachment, medium was replaced with Capan-2 CM, sNF96.2 CM or with DMEM supplemented with 0.5% FBS. Cells were treated or not with 10 μM of SB-431542 TβRI inhibitor. After 11 days of culture at 37 °C under 5% (v/v) CO_2_ atmosphere, cells were fixed with cold methanol and stained with a 0.1% crystal violet solution. After several washes, plates were dried and imaged. Each well was photographed and subsequently analyzed using the ImageJ software (area calculation of Capan-2 cells colonies). These experiments were repeated at least four times.

### Soft agar assay

Capan-2 cells were harvested by trypsinization and diluted to a density of 40,000 cells per mL. Each mL was then suspended in 1 mL of 0.45% melted agarose (Lonza) in culture medium supplemented with FBS 5%, containing antibiotics, and plated onto 6-well plates overlayed with 0.75% agarose in the same medium. The following day, 500 μL of Capan-2 CM (used as control condition) or sNF96.2 CM were added to each well, and cells were treated or not with 10 μM SB-431542 TβRI inhibitor. Cells were then incubated for 3 weeks at 37 °C under 5% (v/v) CO_2_ atmosphere. Renewal of CM and treatments was performed once a week. These experiments were repeated at least three times in triplicate, and twenty fields per condition/per experiment were acquired and subsequently analyzed using the ImageJ software (calculation of the number of Capan-2 cells colonies).

### Statistical analysis

Prism 6.0 (Graphpad) was used for statistics and to create graphs. All data are representative of at least three independent repeats if not otherwise stated. The letter *n* refers to the number of independently performed experiments representative of the data shown in the figures. The statistical significance in this study was determined by two-tailed Student’s *t*-test at *P < 0.05, ** *P* < 0.01, and ***P *<* 0.001. The error bars represent the standard deviation of the mean (SD). To perform the correlation analysis between pSMAD2 pattern and the perineural invasion in human PDAC, a Chi^2^ test was used with a 95% confidence interval. A *p*-value < 0.05 was considered to be significant. Α risk increase was corrected using the Holm–Bonferroni method.

### Human PDAC cohort

All of the samples from patients having undergone surgery in the Hospices Civils de Lyon between the 1st of January 2004 and the 31st of December 2017 for pancreatic ductal adenocarcinoma were included in the cohort. The histopathological slides were reviewed by two pathologists including one expert in pancreatic diseases, blinded to the immunohistochemical data, to assess the presence of perineural invasion. The presence of perineural invasion was defined as either a direct infiltration of the nerves by tumor cells, or when >75% of the nerve was surrounded by tumor cells. These criteria were used to distinguish tumors with perineural invasion (at least one) from those without. This reviewing process also allowed us to select the most representative formalin-fixed paraffin-embedded (FFPE) samples in order to conduct immunohistochemical analyses.

### Immunohistochemical analysis of human PDAC samples

FFPE histological sections were obtained following standard protocols to perform immunohistochemical staining (Ventana Ultra Benchmark) for pSMAD2 (1:100; clone 138 d4, S465/467). Antibody was detect using a peroxidase reaction. A positive control was present on each slide for pSMAD2 (stroma reaction and native acinar pancreatic tissue). pSMAD2 nuclear staining was reviewed by two pathologists including one expert in pancreatic diseases, blinded to the morphological data. pSMAD2 status was considered to be high or low in comparison with fibroblastic cells and acinar pancreatic tissue when the expression was at least equal to or less intense than the controls, respectively.

### Ethics

Human data were obtained from a certified database (MR004 certification no. 19-088), having received a favorable review from the Committee for the protection of persons (CPP) of the Hospices Civils de Lyon (no. 19-109).

### Genetic analyses

For copy number variation analysis (CNV), copy number abnormalities were assessed in the three Schwann cell lines using a high resolution Cytoscan HD single-nucleotide polymorphism array (Affymetrix, santa Clara, CA, USA). The hybridization, amplification and labeling protocols were performed according to the manufacturer’s recommendations (Affymetrix).

For Next Generation Sequencing (NGS), the Illumina MiSeq technology was used for *NF1* mutation screening, alongside the AmpliSeq for Illumina Library Prep (Illumina) for library construction. Paired-end sequencing was performed on the Illumina MiSeq (Illumina) using 300 cycle (Miseq Reagent Nano v2) kit format. Raw signal data were analyzed using in-house bioinformatics pipeline and visualized with the Integrative Genomics Viewer (IGV).

## Results

### Schwann cells induce TGFβ-dependent migration of pancreatic cancer cells

**Fig. 1:**
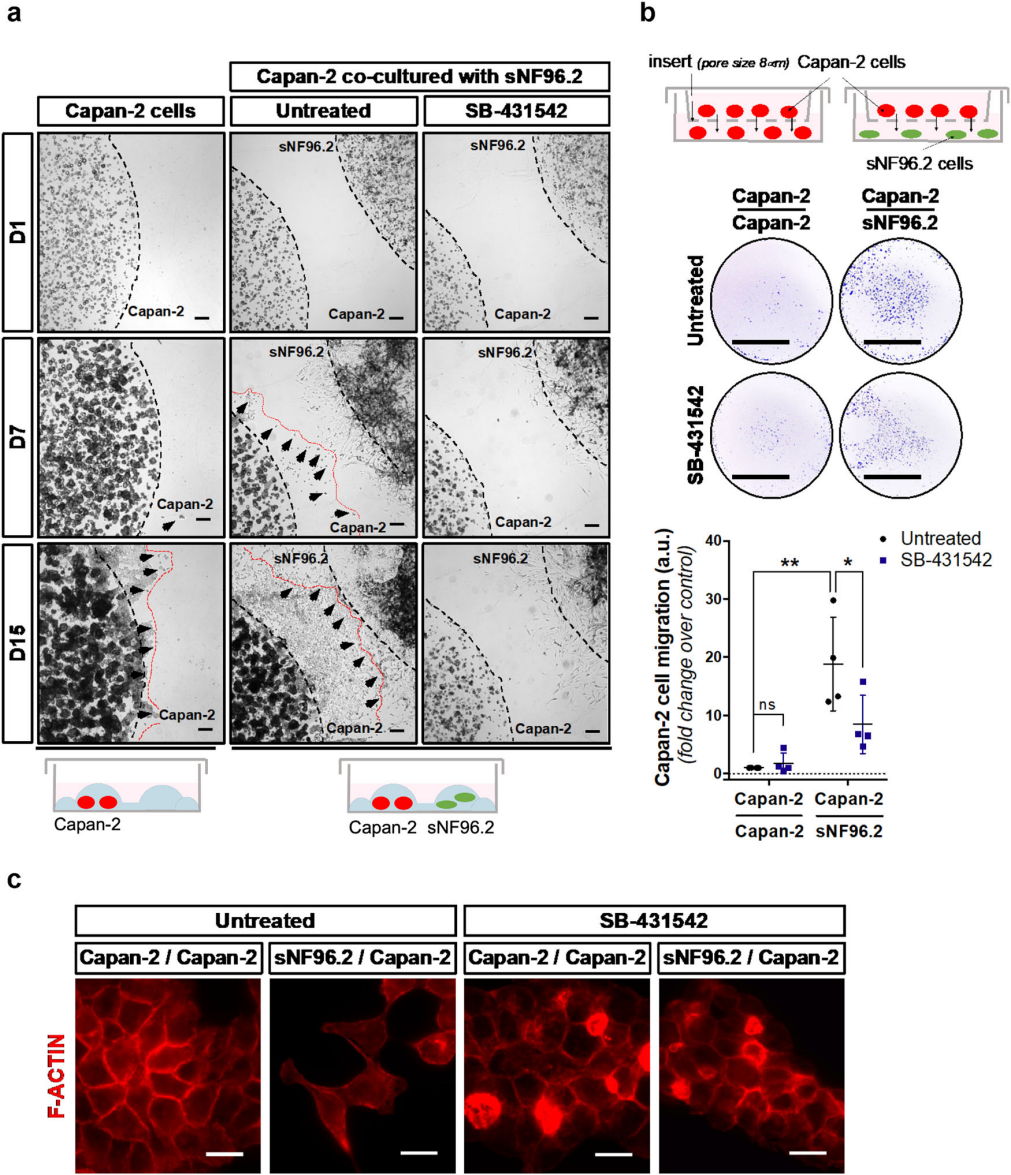
sNF96.2 Schwann cells promote TGFβ-dependent motility of Capan-2 pancreatic cancer cells. **a** Three-dimensional motility assay of Capan-2 cells cultured in matrigel drops alone or in combination with sNF96.2 cells (see schematic diagram below the right field pannels), treated or not with TGFβ type I-receptor (TβRI) inhibitor SB-431542 for 15 days. The black dotted lines indicate the cell localization at the beginning of the experiment. The red dotted lines and the black arrows represent the Capan-2 cell migration front. Bright field images at days 1 (D1), 7 (D7) and 15 (D15) are representative of one experiment performed three times. Scale bars, 200 μm. **b** Boyden chamber migration assay of Capan-2 cells cultured for 72 h alone (Capan-2/Capan-2 condition) or with sNF96.2 cells (Capan-2/sNF96.2 condition), treated or not with SB-431542, and stained with a 0.1% Crystal violet solution. For each condition, an image from one experiment representative of four independent experiments is shown (left panel) and Capan-2 cells migration quantification is represented as mean ± SD (right panel, *n* = 4 independent experiments, *P *<* 0.05; **P *<* 0.01; ns: not significant). Scale bars, 0.5 cm. **c** Direct fluorescence detection of cytoskeletal F-ACTIN by phalloidin-TRITC staining (red) in Capan-2 cells cultured for 24 h, alone (Capan-2/Capan-2 condition) or with sNF96.2 cells (sNF96.2/Capan-2 condition) and treated or not with SB-431542. Representative areas of the images (white frames) are enlarged in the upper right-hand corner of each image. Scale bars, 15 μm.

We first investigated whether Schwann cells could stimulate the motility of pancreatic cancer cells. To achieve this, we performed 3D migration assays to evaluate the orientated migration of Capan-2 pancreatic cancer cells towards sNF96.2 Schwann cells. Capan-2 cells were cultured in a 3D extracellular matrix (ECM) gel drop connected by an ECM gel bridge either to an sNF96.2 cell-containing gel drop or to an empty gel drop (Fig. 1a). Under these conditions, the two cell lines were distinguishable by their shape, Capan-2 cells exhibiting a spherical morphology, while sNF96.2 cells were elongated (Fig. S1). In the presence of sNF96.2 cells, Capan-2 cells clearly migrated further, they entered the ECM gel bridge after 7 days of co-culture with sNF96.2, and had totally invaded the ECM gel after 15 days. Conversely, in the absence of sNF96.2 cells, Capan-2 cells hardly migrated. Of note, sNF96.2 cells also presented unchanged inherent motility properties both in the absence of Capan-2 cells (Fig. S2). We then assessed whether this sNF96.2-induced effect was TGFβ-dependent, and showed that SB-431542, a potent cell-permeable and selective inhibitor of the TGFβ type I-receptor (TβRI), abrogated Capan-2 cancer cell migration towards sNF96.2 cells (Fig. 1a).

Next, using a transwell migration assay (Boyden chamber assay) (Fig. 1b), we observed a significant increase in the migration of Capan-2 cells (upper chamber) co-cultured with sNF96.2 cells (lower chamber) rather than with Capan-2 cells (fold change: 18.85). We then confirmed that Capan-2 cell migration was TGFβ-dependent, as the addition of SB-431542 significantly decreased their migration towards sNF96.2 cells (fold change: 8.45) in co-culture experiments (Fig. 1b). As a control, we observed that TGFβ added to the Capan-2 culture induced the migration of these cells, which was compromised by SB-431542 (Fig. S3a).

In addition, fluorescent detection of cytoskeletal actin (Fig. 1c) revealed that Capan-2 cells co-cultured with sNF96.2 cells (i.e., sNF96.2/Capan-2 condition) acquired an elongated shape, displaying actin reorganization compared to the control condition (i.e., Capan-2/Capan-2 condition), in which Capan-2 cells formed compact and cohesive colonies. This plasticity in the shape of Capan-2 cells, enhanced by the co-culture with sNF96.2 cells, was lost upon exposure to SB-431542 (Fig. 1c).

sNF96.2 cells are derived from a patient with type 1 neurofibromatosis (NF1). It is widely recognized that neurofibroma development in NF1 depends on a somatic *NF1* mutation in a Schwann cell, rendering the cell deficient in *NF1*-encoded tumor suppressor protein neurofibromin function. NF1-null peripheral nerve sheath tumors have been demonstrated to express elevated levels of TGFβ^40^. Loss of NF1 was also reported to increase TGFβ expression in mastocystes^41^. Furthermore, mouse *Nf1*^−/−^ nerves contain increased levels of TGFβ^42^. Whether the loss of NF1 is actually driving TGFβ secretion in sNF96.2 cells is unknown. Since sNF96.2 cells have been reported to be mutated for *NF1*, we designed experiments using two other types of Schwann cells not previously reported to be mutated for *NF1*, namely HSwC (human primary Schwann cells isolated from human spinal nerve) and ipn02.3 2λ (immortalized patient derived plexiform neurofibroma Schwann cells). We first determined the genetic status of these cells. Copy number variation (CNV) and next generation sequencing (NGS) analyses showed that HSwC and ipn02.3 2λ display an unmutated status for *NF1* (Fig. S4). Conversely, and as expected from the literature, sNF96.2 cells presented an allele deleted for *NF1* and a mutation on the other allele (Fig. S4). We further observed using a transwell migration assay, a significant increase in the migration of Capan-2 cells (upper chamber) co-cultured with HSwC and ipn02.3 2λ cells (lower chamber) rather than with Capan-2 cells in the same proportion to that observed with sNF96.2 cells (Fig. S5a). In all co-culture conditions, migration was compromised by SB-431542 treatment. These results indicate that the TGFβ-dependent increase in migration of Capan-2 cells cultured in the presence of sNF96.2 cells does not result from the loss of NF1.

Collectively, these results demonstrate that sNF96.2 cells promote the motility and reshaping of Capan-2 cells through a TGFβ-dependent mechanism.

### Schwann cells activate the TGFβ-SMAD signaling pathway in Capan-2 cancer cells

**Fig. 2:**
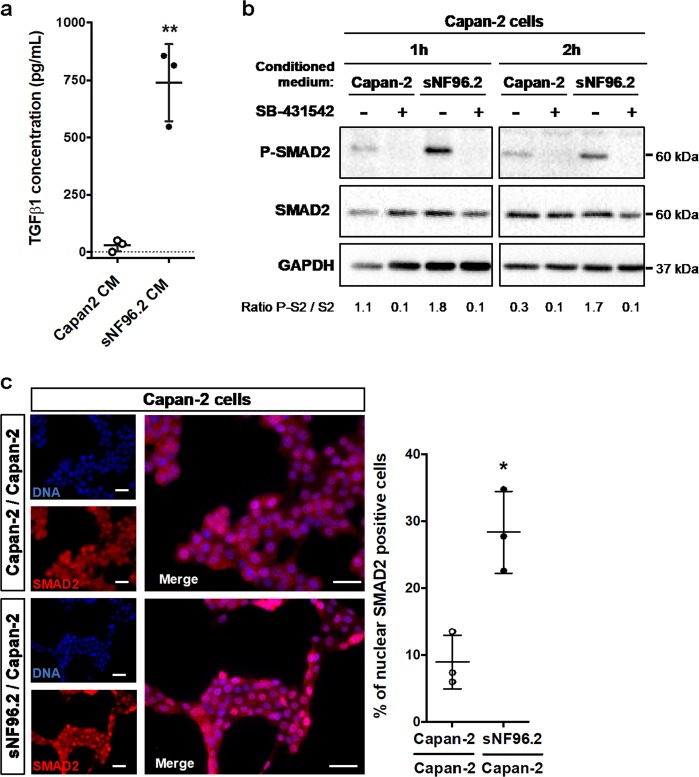
sNF96.2 cells secrete TGFβ and stimulate the SMAD signaling pathway in Capan-2 cells. **a** Amount of soluble TGFβ1 secreted into the medium by Capan-2 cells (Capan-2 CM) and by sNF96.2 cells (sNF96.2 CM). Quantification of soluble TGFβ1 concentration is represented as mean ± SD (*n* = 3 independent biological replicates, **P *<* 0.01). **b** Western blot detection of phospho-SMAD2 protein (P-SMAD2) in Capan-2 cells cultured for 1 h or 2 h either with Capan-2 CM or with sNF96.2 CM, and treated or not with the TβRI inhibitor, SB-431542. GAPDH and total SMAD2 were used as loading controls. Ratio of phospho-SMAD2 to total SMAD2 levels are indicated below the blots. **c** Immunofluorescence detection of SMAD2 protein (red) in Capan-2 cells cultured for 2 h alone (Capan-2/Capan-2 condition) or with sNF96.2 cells (sNF96.2/Capan-2 condition). Cell nuclei were counterstained with DAPI (blue). Scale bars, 30 μm. For each condition, images from one experiment representative of three independent experiment are shown (left panel) and quantification of nuclear SMAD2 per total number of nuclei is represented as mean ± SD (right panel, *n* = 3 independent experiments, *P *<* 0.05). *CM* conditioned medium.

To test the hypothesis that sNF96.2 cells represent a source of TGFβ, we performed ELISA tests, measuring the amounts of TGFβ secreted into the conditioned medium (CM) of both Capan-2 cells and Schwann cells. sNF96.2 cells secreted significantly higher amounts of TGFβ1 compared to Capan-2 cells (sNF96.2 CM ~850 pg/mL vs. Capan-2 CM ~50 pg/mL) (Fig. 2a). We also observed that NF1-positive HSwC and ipn02.3 2λ cells secreted higher amounts of TGFβ (Fig. S5b). TGFβ is a secreted polypeptide that belongs to a large family encompassing cytokines and growth factors including TGFβs, Bone Morphogenetic Proteins (BMPs) and Activins. TGFβ signals through heterodimeric serine/threonine-kinase receptor complexes, constituted of TβRII and TβRI proteins^43,44^. After binding to its receptor complex, TGFβ induces the phosphorylation of TβRI, which phosphorylates SMAD2 and SMAD3 proteins in their C-terminal regions (P-SMAD2/3), leading to their interaction with the SMAD4 protein. P-SMAD2/3-SMAD4 complexes accumulate within the nucleus, bind to DNA and enhance the transcription of target genes. We thus investigated whether this TGFβ-enriched CM from sNF96.2 cells could promote TGFβ-SMAD signaling pathway activation in Capan-2 cells. We performed Western blot experiments, which revealed a clear increase in the phosphorylated form of SMAD2 in Capan-2 cells treated for 1 h or 2 h with sNF96.2 CM (Fig. 2b), as well as a complete impairment in SMAD2 phosphorylation in the presence of SB-431542. Finally, we showed by immunofluorescence a significant nuclear accumulation of SMAD2 in Capan-2 cells co-cultured with sNF96.2 cells (Fig. 2c).

Altogether, these results highlight that sNF96.2 cells secrete TGFβ, leading to the activation of the SMAD signaling pathway in Capan-2 cells.

### Conditioned medium from Schwann cells is sufficient to induce pancreatic cancer cell migration

**Fig. 3:**
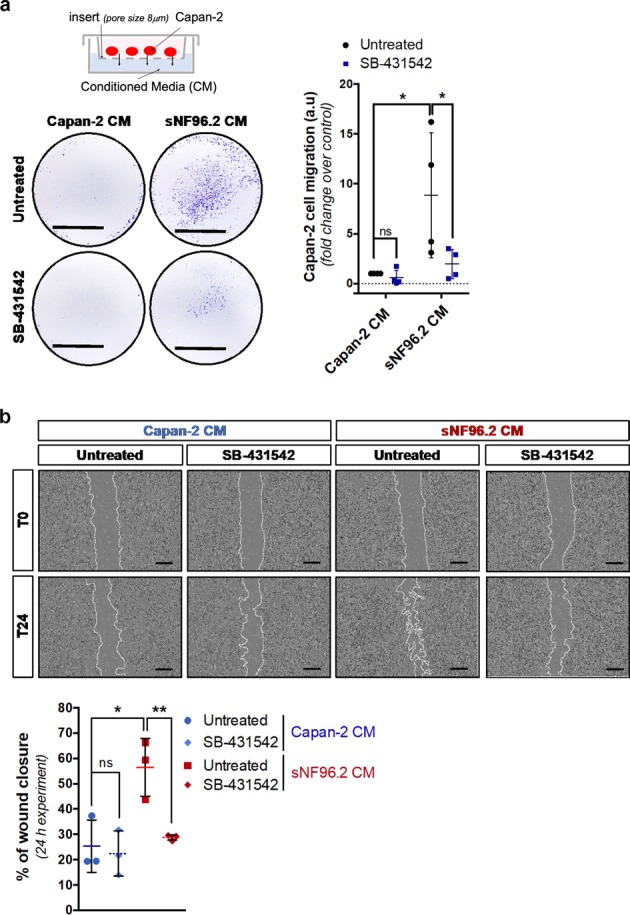
Secreted factors from sNF96.2 cells stimulate Capan-2 cell migration in a TGFβ-dependent manner. **a** Boyden chamber migration assay of Capan-2 cells cultured for 72 h either with Capan-2 CM or with sNF96.2 CM, treated or not with the TβRI inhibitor, SB-431542, and stained with a 0.1% crystal violet solution. For each condition, an image from one experiment representative of four independent experiments is shown (left panel) and Capan-2 cell migration quantification is represented as mean ± SD (right panel, *n* = 4 independent experiments, *P *<* 0.05; ns: not significant). Scale bars, 0.5 cm. **b** Wound healing assay of Capan-2 cells cultured for 24 h either with Capan-2 CM (blue color code) or with sNF96.2 CM (red color code), treated or not with SB-431542. Bright field images at time 0 (T0) and 24 (T24) h are representative of one experiment performed three times (upper panel) and quantification of wound closure at 24 h is represented as mean ± SD (bottom panel, *n* = 3 independent experiments, *P *<* 0.05; **P *<* 0.01*;* ns: not significant). Scale bars, 400 μm. *CM* conditioned medium.

To test whether factors, including TGFβ, secreted by Schwann cells were sufficient to enhance the migration of Capan-2 cells, we performed Capan-2 cell migration assays in Boyden chambers, using either conditioned medium from sNF96.2 (sNF96.2 CM) or Capan-2 (Capan-2 CM) cells in the lower compartment (Fig. 3a). We observed that CM from Capan-2 cells used as a control, had no significant effect, whereas CM from sNF96.2 cells significantly stimulated Capan-2 cell migration (fold change: 8.85). In addition, we observed that TGFβ-signaling inhibition by SB-431542 significantly abrogated this effect (fold change: 1.95). Wound healing assays further confirmed the ability of sNF96.2 CM to promote Capan-2 cell migration (24 h wound closure, sNF96.2 CM: 56.48% vs. Capan-2 CM: 25.35%), as well as the involvement of TGFβ activation in this process as demonstrated by the significant decrease in the sNF96.2 CM-induced effect, upon exposure to SB-431542 (24 h wound closure, sNF96.2 CM + SB-431542: 28.73% vs. sNF96.2 CM: 56.48%) (Fig. 3b). As a control, we validated the ability of SB-431542 to abrogate TGFβ-enhanced wound closure in Capan-2 cells (Fig. S3b). This result was validated using CM from NF1-positive HSwC and ipn02.3 2λ cells that also induced TGFβ-dependent migration of Capan-2 cells (Fig. S5c). These data reveal that conditioned medium from sNF96.2 cells is sufficient to promote the migration of Capan-2 cells in a TGFβ-dependent manner.

**Fig. 4:**
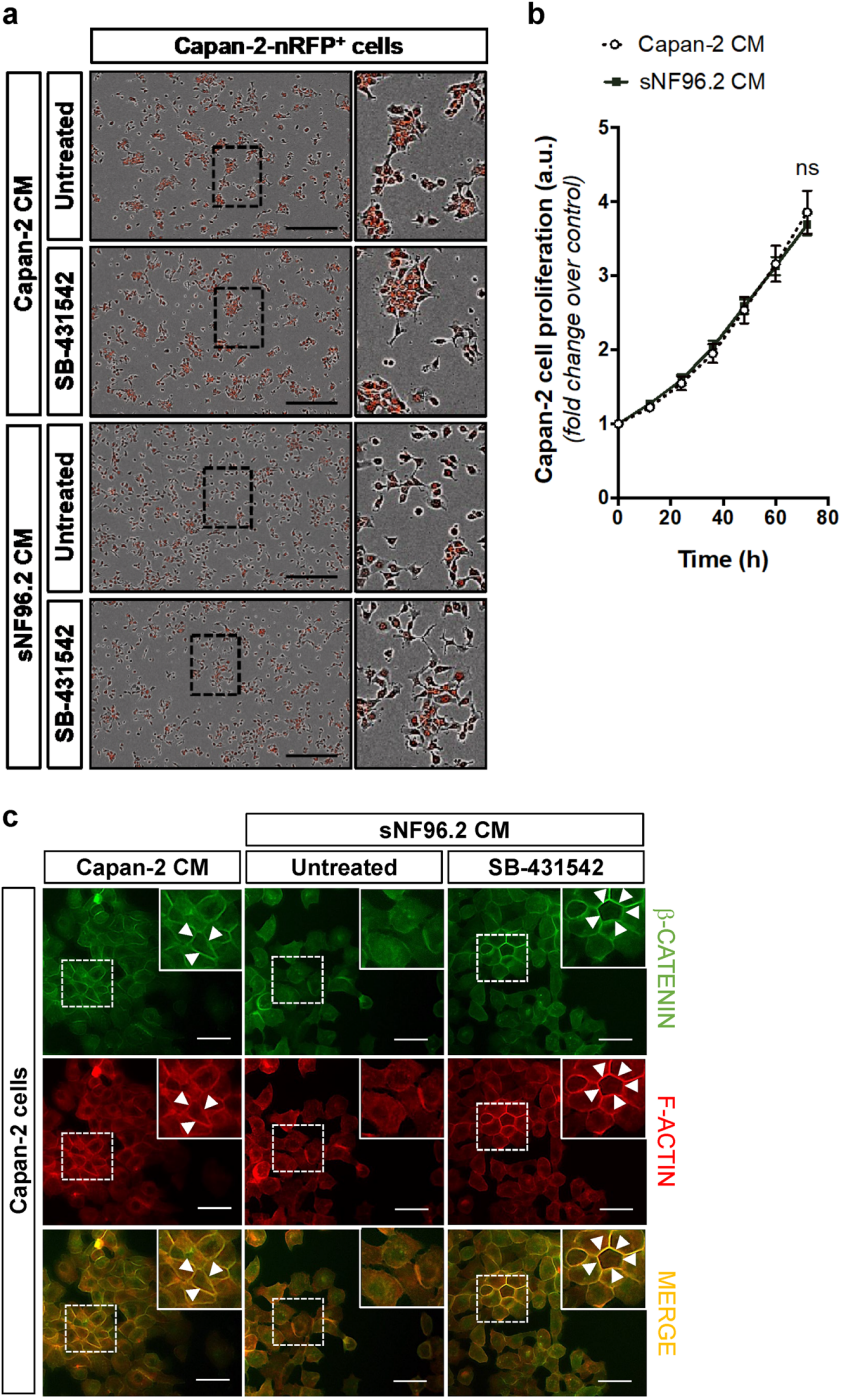
Secreted factors from sNF96.2 cells modulate Capan-2 cell–cell interactions in a TGFβ-dependent manner. **a** Images of fluorescent Capan-2-nRFP^+^ cells cultured for 48 h either with Capan-2 CM or with sNF96.2 CM, and treated or not with SB-431542 TβRI inhibitor (IncuCyte™ Zoom imaging system). The black frames show representative areas of Capan-2 cells enlarged in the right-hand side of each image. Scale bars, 400 μm. **b** Proliferation assay of Capan-2-nRFP^+^ cells cultured for 72 h either with Capan-2 CM (white dot) or with sNF96.2 CM (black square) (automated live cell time-lapse counting using an IncuCyte™ Zoom imaging system). Quantification of Capan-2 cell proliferation is represented as mean ± SD (*n* = 3 independent experiments, significance is shown for the 72-h timepoint, ns: not significant). **c** Immunofluorescence detection of F-ACTIN (red) and β-CATENIN (green) proteins in Capan-2 cells located at the migratory front in a wound healing assay after 24 h, cultured either with Capan-2 CM or with sNF96.2 CM and treated or not with SB-431542. The white frames show representative areas that are enlarged in the upper right-hand corner of each image. White arrowheads in the insets show the plasmic membrane. Scale bars, 15 μm. *CM* conditioned medium.

We next sought to characterize the cancer cell phenotype associated with Schwann cell-mediated migratory functions. We generated a stable Capan-2 cell line expressing the mKate nuclear Red Fluorescent Protein (*i.e*. Capan-2-nRFP^+^ cells). Using fluorescent live cell imaging (Incucyte® system), we observed that, in comparison with control Capan-2 CM, sNF96.2 CM enhanced Capan-2-nRFP^+^ cell spreading and dispersion (Fig. 4a). Indeed, the Capan-2 cells treated with sNF96.2 CM, grew as individualized and non-cohesive cells, exhibiting an elongated shape as initially observed in co-culture experiments (Fig. 1c). Once again, the addition of SB-431542 decreased this sNF96.2 cell-induced effect, supporting the active role of TGFβ in the regulation of Capan-2 cell behavior (Fig. 4a). We also observed that proliferative properties of Capan-2 cells were unchanged in both conditions (with sNF96.2 CM or with Capan-2 CM) (Fig. 4b), ruling out the possibility that the increased dispersion of the Capan-2-nRFP^+^ cells treated with the sNF96.2 CM resulted from an increased number of cells. Hence, these results demonstrate that Capan-2 cells undergo a TGFβ-dependent cell-shape remodeling in response to factors secreted by Schwann cells. In order to better characterize these morphological changes, we analyzed two cell–cell junction markers, β-CATENIN and F-ACTIN, in Capan-2 cells subjected to wound healing assays in the presence of sNF96.2 CM (Fig. 4c). We observed that sNF96.2 CM induces a decrease in β-CATENIN and F-ACTIN membrane staining in Capan-2 cells which were at the edge of the wound, illustrating a reduction in their cell–cell cohesion. Furthermore, addition of SB-431542 was correlated with a better conservation of these membrane markers, validating the involvement of TGFβ in this sNF96.2 CM-induced effect. We obtained similar results in co-culture experiments (β-CATENIN and E-CADHERIN relocalization) (Fig. S6), reinforcing our previous observation with F-ACTIN (Fig. 1c), and confirming that Capan-2 cell dispersion is promoted by Schwann cells.

Hence, the TGFβ-dependent migratory properties bestowed to Capan-2 cells by Schwann cells are associated with impaired cell–cell junctions.

### Schwann cells stimulate the oncogenic potential of pancreatic cancer cells

**Fig. 5:**
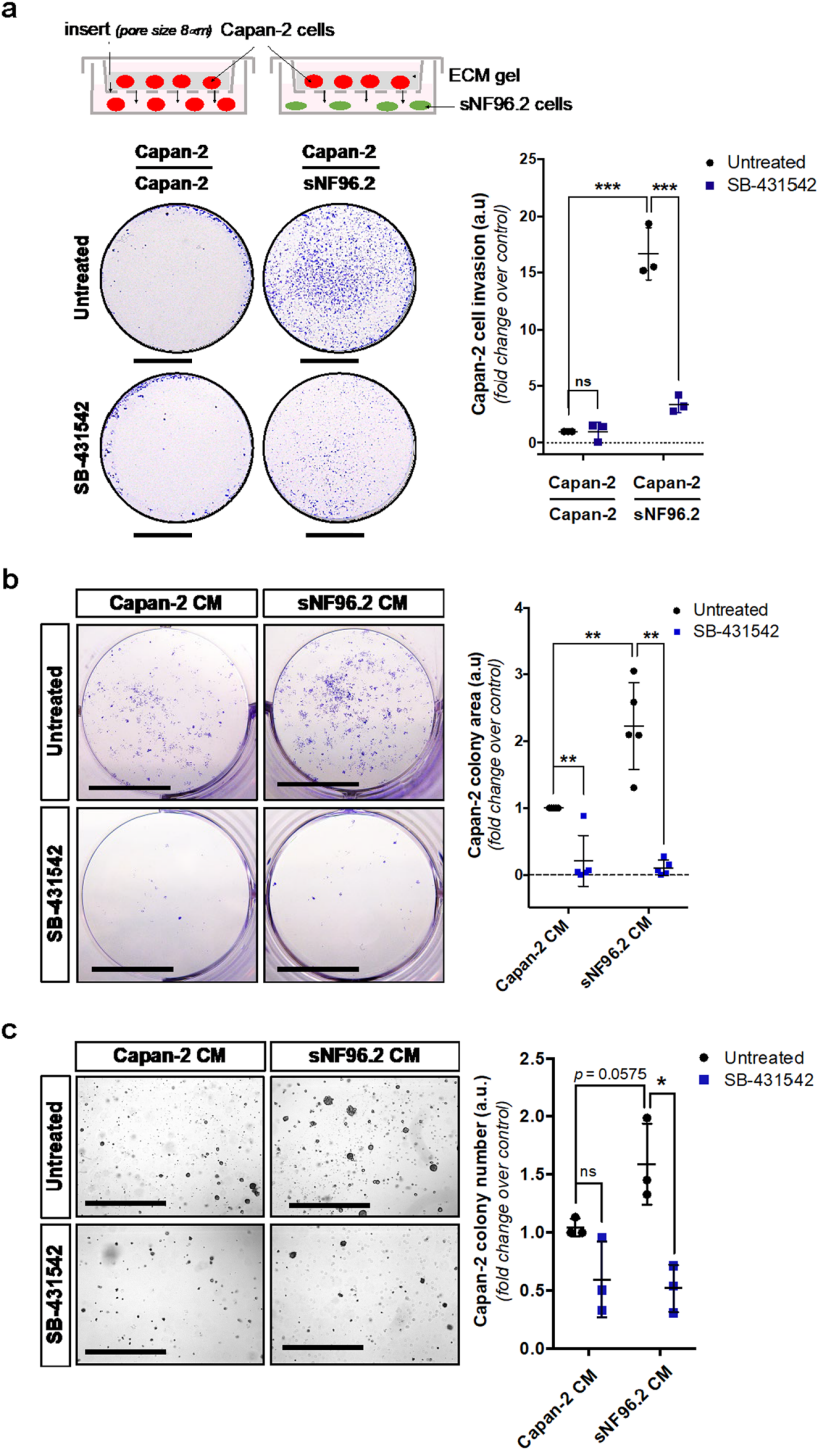
sNF96.2 cells promote TGFβ-dependent oncogenic properties of Capan-2 cells. **a** Boyden chamber invasion assay in matrigel of Capan-2 cells cultured for 72 h alone (Capan-2/Capan-2 condition) or with sNF96.2 cells (Capan-2/sNF96.2 condition), treated or not with the TβRI inhibitor, SB-431542, and stained with a 0.1% crystal violet solution. For each condition, an image from one experiment representative of three independent experiments is shown (left panel) and Capan-2 cell invasion quantification is represented as mean ± SD (right panel, *n* = 3 independent experiments, ***P *<* 0.001; ns: not significant). Scale bars, 0.5 cm. **b** Two-dimensional clonogenic survival assay of Capan-2 cells seeded at low density and cultured for 11 days either with Capan-2 CM or with sNF96.2 CM, treated or not with SB-431542 and stained with a 0.1% crystal violet solution. For each condition, an image from one experiment representative of five independent experiments is shown (left panel) and Capan-2 colony area quantification is represented as mean ± SD (right panel, *n* = 5 independent experiments, **P *<* 0.01). Scale bars, 0.5 cm. **c** Anchorage-independent cell proliferation by soft agar assay of Capan-2 cells cultured for 3 weeks either with Capan-2 CM or with sNF96.2 CM and treated or not with SB-431542. For each condition, an image from one experiment representative of three independent experiments is shown (left panel) and Capan-2 colony number quantification is represented as mean ± SD (right panel, *n* = 3 independent experiment, *P *<* 0.05; ns: not significant). *CM* conditioned medium. Scale bars, 0.1 cm.

Since such migratory properties are associated with aggressive tumor cells, we wondered whether this modulation of cellular behavior, induced by Schwann cells, was correlated with increased tumorigenesis of pancreatic cancer cells. We first investigated the invasive properties of Capan-2 cells, as a feature of their tumorigenic potential. Capan-2 cells displayed a greater ability to migrate and invade the ECM gel in a transwell assay when co-cultured with sNF96.2 cells rather than Capan-2 cells (fold change: 16.66) (Fig. 5a). Furthermore, the addition of SB-431542 induced a five-fold decrease in the invasive abilities of Capan-2 cells (fold change: 3.38). Of note, we also showed the ability of SB-431542 to abrogate TGFβ-enhanced invasive abilities of Capan-2 cells (Fig. S7). These observations demonstrate that Schwann cells stimulate TGFβ-dependent invasion of pancreatic cancer cells.

In order to determine whether conditioned medium from sNF96.2 cells modulates Capan-2 cell survival, another hallmark of aggressive cancer cells, we performed 2D colony formation assays over an 11-day experimental time course. We observed that CM from sNF96.2 cells supports the survival of Capan-2 cells seeded at a low density with a two-fold increase in the final colony area (fold change vs. control: 2.22). Moreover, colony formation in the presence of both CM from Capan-2 and sNF96.2 cells, was blocked after the addition of SB-431542 (fold change, Capan-2 CM + SB-431542: 0.2; sNF96.2 CM + SB-431542: 0.1) (Fig. 5b). Finally, using soft agar assays we observed an increase in the ability of Capan-2 cells to grow as anchorage-independent colonies when treated with sNF96.2 CM (compared to Capan-2 CM) for 3 weeks (fold change: 1.98), highlighting their oncogenic potential (Fig. 5c). This effect was reduced by adding the TβRI inhibitor (fold change vs. control: 0.7). These results thus indicate the in vitro ability of Schwann cells to enhance invasive properties of Capan-2 cells, as well as their ability to survive and grow as transformed cells.

**Fig. 6:**
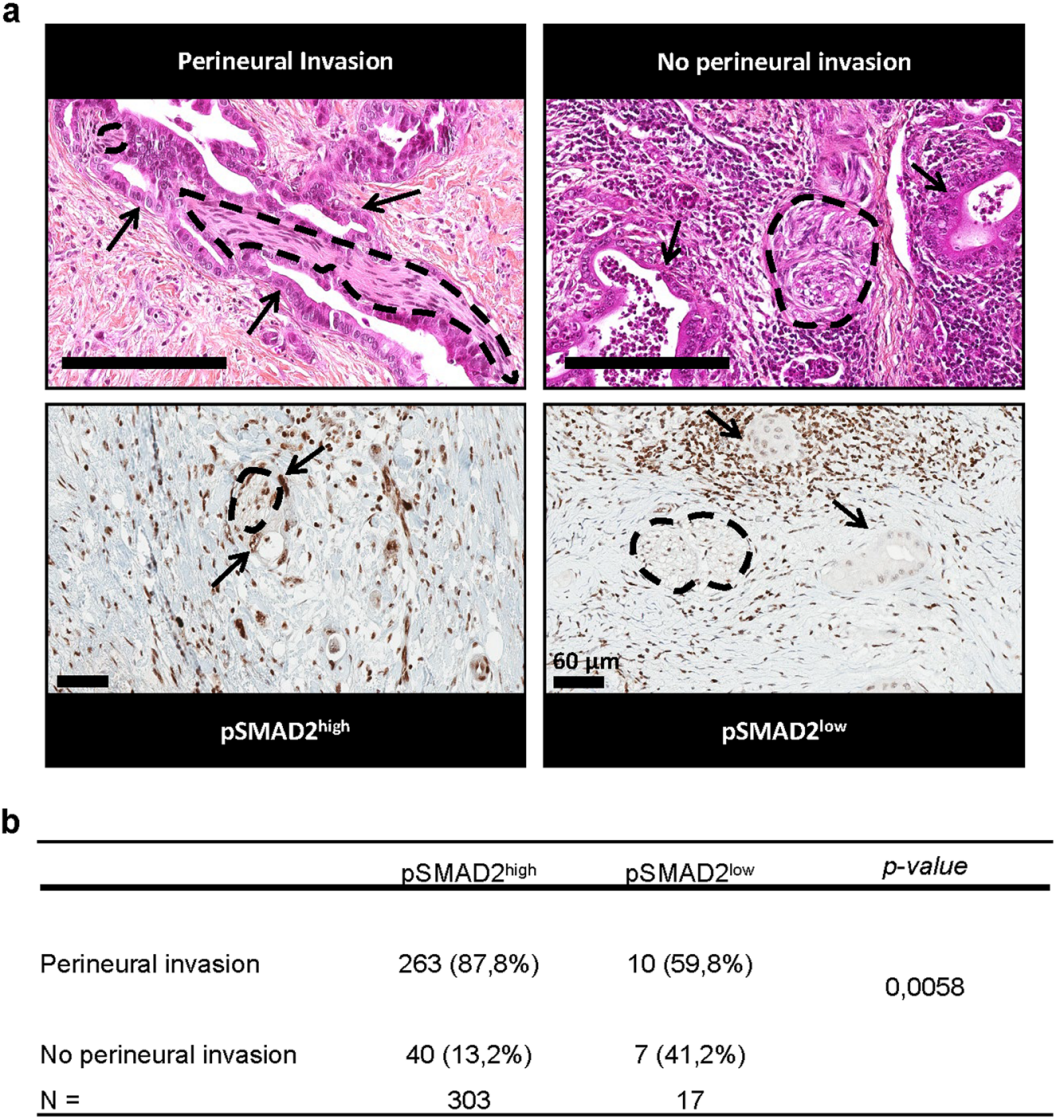
pSMAD2 expression in human moderately differentiated PDAC and its association with perineural invasion. **a** Hematoxylin and eosin (top images; scale bars, 200 μm) and pSMAD2 immuno histochemistry (bottom images; scale bars, 60 μm) of a representive patient with perineural invasion and a patient with no perineural invasion. Nerves are highlighted with dotted lines and tumor cells with arrows. **b** Distribution of tumors depending on the presence of perineural invasion and the pSMAD2 level.

We next explored TGFβ signaling activation in 320 moderately differentiated human PDAC tumors (Supplementary data 1). We analyzed by IHC the pSMAD2 expression (used as a readout of TGFβ signaling activation) and explored the relationship between tumor cells and nerves. In vivo, pathologists define the perineural invasion as the penetration of tumor cells within nerve sheats. Inversely, when no peurineural invasion occurs, tumor cells remain at a distance from nerves (Fig. 6a upper right-hand panel). Our analysis revealed two pSMAD2 expression patterns (Fig. 6a lower panels): pSMAD2^high^, corresponding to a staining instensity at least equal or superior to that of the surrounding stromal and acinar cells; pSMAD2^low^, corresponding to a staining intensity less intense than that of the surrounding stromal and acinar cells. Among the 320 PDAC samples, 303 were identified as pSMAD2^high^, and 17 as pSMAD2^low^ (Fig. 6b). Importantly, we found a significant statistical association between the presence of perineural invasion and the pSMAD2^high^ status (Fig. 6b; *P* = 0.0058). This result strongly suggests that high activation of the SMAD TGFβ-signaling pathway is positively correlated with perineural invasion in PDAC.

### Identification of secreted proteins involved in pancreatic cancer cell motility by mass spectrometry

**Fig. 7:**
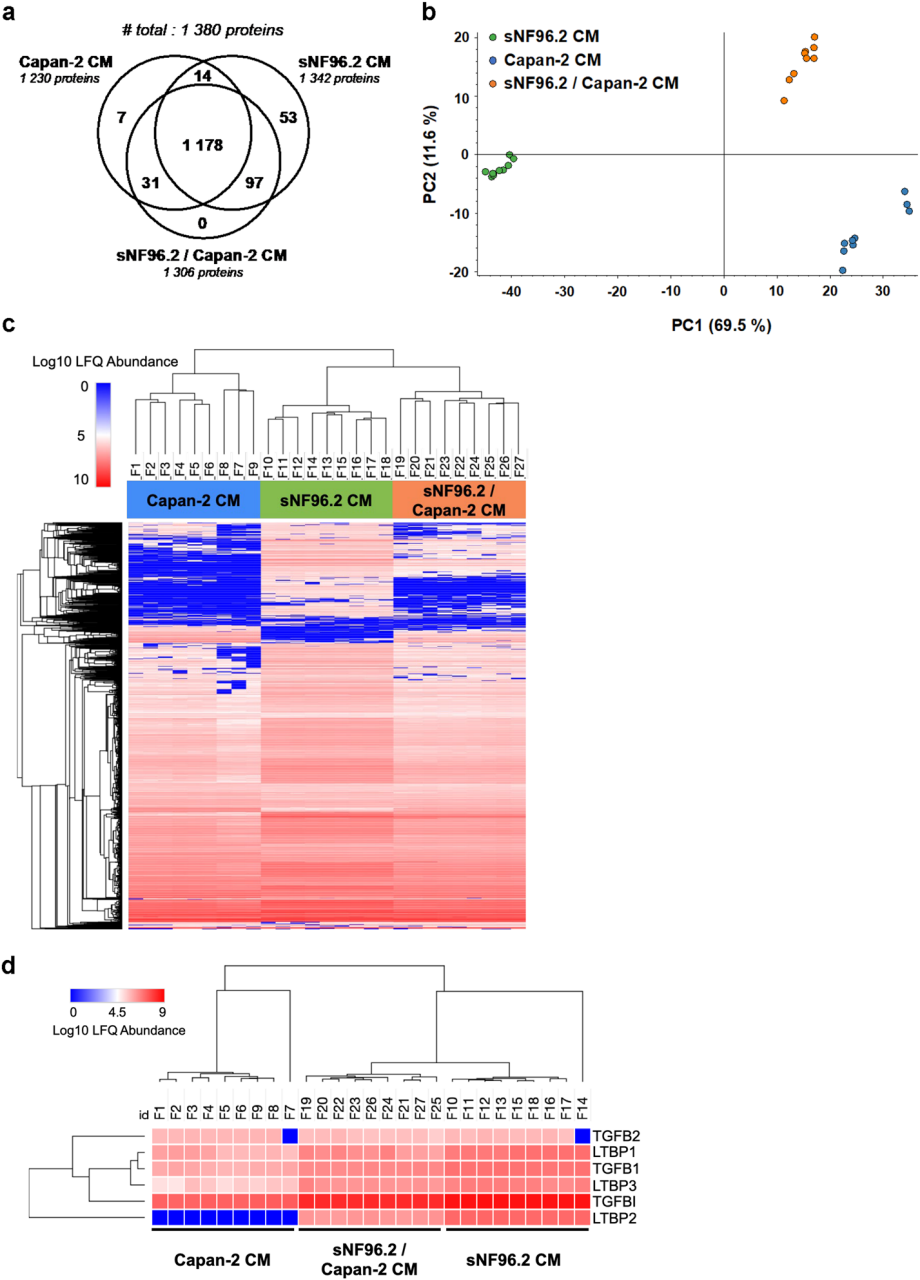
Mass spectrometry analysis of the secretomes of Capan-2 and sNF96.2 cells cultured alone or in combination. **a** Venn diagram showing the number of proteins quantified in Capan-2 CM, sNF96.2 CM and sNF96.2/Capan-2 CM, using LFQ mass spectrometry. **b** Principal component analysis of LFQ mass spectrometry data performed using the Proteome Discover 2.2 software. Each dot represents a CM sample, and the percentages of total variance are shown in brackets. **c** Hierarchical clustering of secreted proteins identified in Capan-2 CM, sNF96.2 CM and sNF96.2/Capan-2 CM. Color code is representative of the log10 normalized LFQ abundance values. **d** Hierarchical clustering of the indicated TGFβ superfamily members in Capan-2 CM, sNF96.2 CM and sNF96.2/Capan-2 CM. Color code is representative of the log10 normalized LFQ abundance values. *CM* conditioned medium, *LFQ* label-free quantitative.

In our effort to obtain a comprehensive picture of the signaling networks between pancreatic cancer cells and Schwann cells, we then determined the proteomic profiles of secretomes from Capan-2 and sNF96.2 cells, cultured alone or in combination (Capan-2 CM, sNF96.2 CM and sNF96.2/Capan-2 CM). Three biological CM replicates were produced and were further analyzed using a label-free quantitative (LFQ) workflow, in three technical replicates (Fig. S8). Raw data were processed with Proteome Discover 2.2, a false discovery rate (FDR) of less than 1% was applied, and a group of 39 proteins close to the detection limit was excluded, allowing the final identification of 1,380 proteins secreted by Capan-2 and sNF96.2 cells cultured alone or in combination (Supplementary data 2). Of these, 1,230, 1,342 and 1,306 were identified in Capan-2 CM, sNF96.2 CM, or Capan-2 /sNF96.2 co-culture CM, respectively (Fig. 7a; Supplementary data 2). A group of 1,178 proteins was secreted in all CM, whereas 7 and 53 proteins were exclusively found in Capan-2 and sNF96.2 CM samples, respectively. No proteins were exclusively found in sNF96.2/Capan-2 CM. Regarding their relative LFQ abundance, the three secretomes exhibited distinct profiles. Indeed, principal component analysis (Fig. 7b) and hierarchical clustering (Fig. 7c) revealed that biological and technical replicates of each CM clustered together and distinctly from other CM. We then examined the relative abundance of TGFβ family members identified within the three secretomes. Of note, the TGFβ-induced protein (TGFβI), the latent forms of the three TGFβ isoforms (LTBP1, LTBP2 and LTBP3), and the active form of TGFβ1 were more abundant in CM from sNF96.2 cells and from co-cultures, in comparison with CM from Capan-2 cells alone, as illustrated in the hierarchical clustering heatmap (Fig. 7d). Altogether, these mass spectrometry results reveal that Capan-2 and sNF96.2 cells, cultured alone or in combination exhibit distinct secretome patterns.

**Fig. 8:**
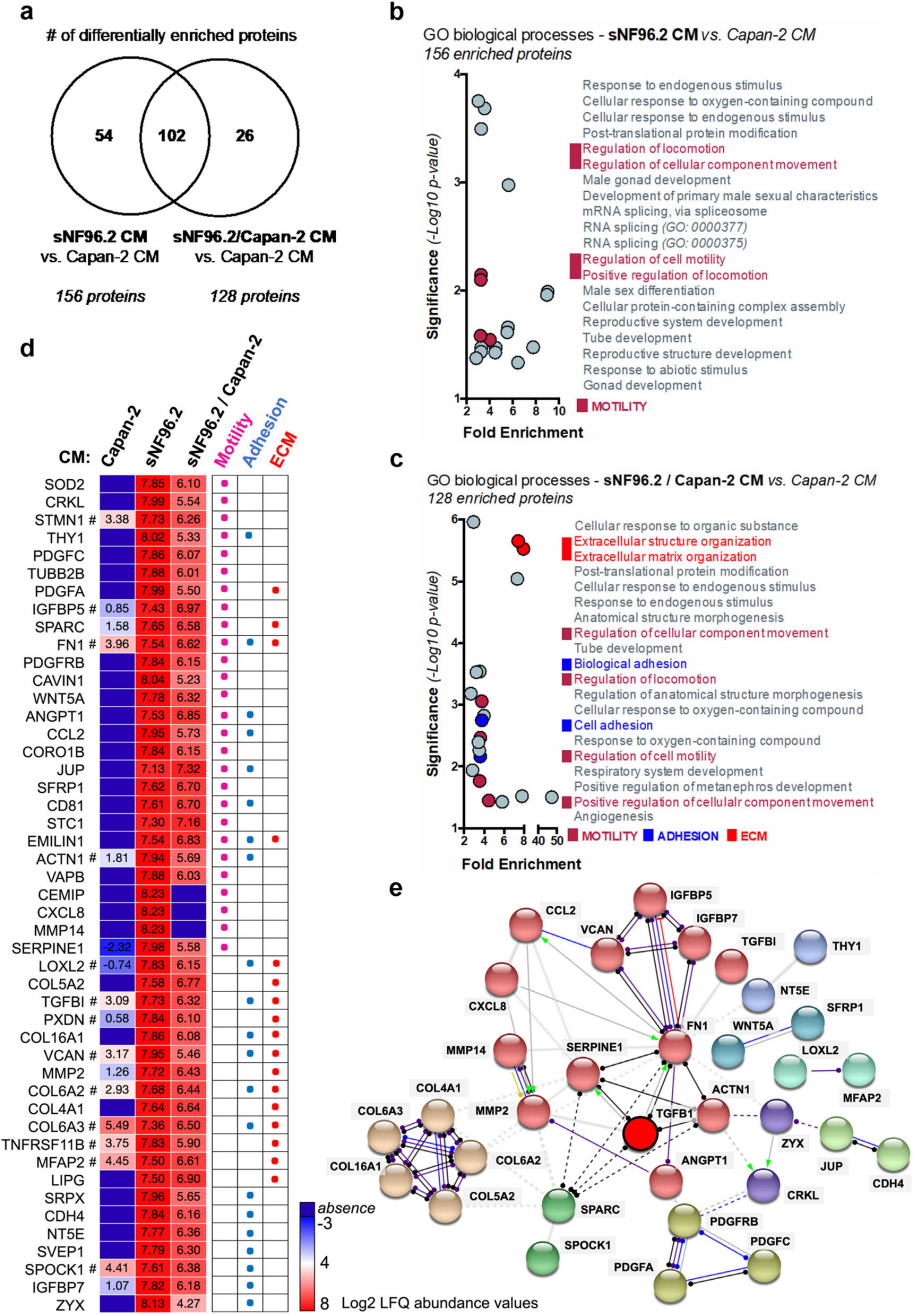
Biological processes correlated with proteins more abundant in conditioned medium of sNF96.2 cells and Capan-2 cells cultured alone or in combination. **a** Venn diagram showing the number of significantly differentially enriched proteins quantified (fold change >2; adjusted *p*-value < 0.05) in sNF96.2 CM and sNF96.2/Capan-2 CM compared to Capan-2 CM. **b**, **c** GO enrichment analysis of differentially secreted proteins in sNF96.2 CM (156 proteins) and in sNF96.2/Capan-2 CM (128 proteins), compared to Capan-2 CM. The figures show the top 20 GO biological processes grouped by enrichment (>2-fold) and *p*-value (<0.05). Motility, adhesion and ECM related-groups are represented in different colors. **d** Heatmap depicting the abundance levels (log10 normalized LFQ abundance values) of 47 individual proteins identified within motility, adhesion and ECM related-GO biological processes, in the three sNF96.2, Capan-2 and sNF96.2/Capan-2 CM. # are representative of a non-significant difference between sNF96.2 CM and Capan-2 CM. **e** Protein–protein predicted functional associations between 33 proteins enriched in sNF96.2 CM and sNF96.2/Capan-2 CM compared to Capan-2 CM and associated with motility, adhesion and ECM GO groups, are represented by a protein-protein network graph (STRING database analysis). The protein network is separated into 9 clusters (see node colors), in which inter-cluster edges are represented as dashed lines. Inter-node edges are representative both of types of action (color code: red, inhibition; blue, binding; purple, catalysis; black, reaction; green, activation; yellow, transcriptional regulation; gray, unspecified) and effects (unspecified, positive or negative) for predicted interactions between proteins. *CM* conditioned medium, *ECM* extracellular matrix, *GO* gene ontology, *STRING* search tool for the retrieval of interacting genes/proteins.

In order to better understand the effects of Schwann cells on Capan-2 cells, we identified 156 and 128 differentially enriched proteins (fold change > 2; adjusted *p*-value < 0.05) in sNF96.2 CM and sNF96.2/Capan-2 co-culture CM, compared to Capan-2 CM (Fig. 8a; Supplementary data 3). Of these, 54 were specific to sNF96.2 CM, 26 to sNF96.2/Capan-2 CM compared to Capan-2 CM, and 102 were common to secretomes from both sNF96.2 cells and sNF96.2/Capan-2 co-cultures. These groups of enriched secreted factors (i.e., 156 and 128 enriched proteins groups) were further subjected to gene ontology (GO) enrichment analysis (Supplementary data 4). We observed that the proteins upregulated in CM from sNF96.2 cells were involved in cell motility biological processes, including positive regulation of locomotion, regulation of cellular component and regulation of cell motility (Fig. 8b; Supplementary data 4). Interestingly, these cell motility features were also found in CM from sNF96.2/Capan-2 co-culture, along with cell adhesion and extracellular matrix organization processes (Fig. 8c; Supplementary Data 4). We then isolated a group of 47 proteins overrepresented in sNF96.2 and sNF96.2/Capan-2 co-culture secretomes, compared to Capan-2 CM, which were associated with cell motility mechanisms (i.e. *“*motility” GO group) and regulation of cell adhesion properties (i.e. *“*adhesion” and “ECM” GO groups; Fig. 8d). Hence, these results demonstrate that Schwann cells are a source of numerous proteins involved in both cell motility mechanisms and regulation of cell adhesion properties, further strengthening our previous in vitro data. To gain further insight into the integrated protein-protein interactions of these Schwann cell-secreted signaling proteins, we next established a protein interaction network using the STRING database (Fig. 8e). Of note, in order to demonstrate the important involvement of TGFβ signaling within this predicted network, the TGFβ1 factor was added to the 47 previously identified proteins. As such, we identified a total of 63 protein-protein predicted functional associations between 33 assessed proteins, including the TGFβ1 cytokine (protein-protein interaction enrichment *p*-value < 1.0e^−16^). Results highlighted that these proteins were highly inter-connected, and that the TGFβ1 factor exhibited four potential interactions with SERPINE1, FN1, MMP2, and ACTN1 proteins.

Altogether, these data reveal that TGFβ may be connected to the protein network identified as a motility/adhesion pattern resulting from the dialog between Schwann cells and pancreatic cancer cells.

## Discussion

Pancreatic cancer-associated neural remodeling (PANR) is a hallmark of PDAC involved in neuropathic pain, cancer cell dissemination, and local relapse after surgery^9,14^. Identification of molecular factors responsible for PANR represents a major challenge for patient management. Neuroplastic changes occurring during PDAC development are controlled by a multi-directional dialog between cancer cells, nerves and Schwann cells, and other stromal cells. The molecular bases of this intercellular communication network remain largely unknown. In particular, our understanding of the impact of Schwann cells on tumor cells is very poor. In the present study, we demonstrated that sNF96.2 Schwann cells have the ability to enhance aggressiveness of Capan-2 cancer cells (migration, invasion and tumorigenicity) through TGFβ signaling. Indeed, we showed that these effects relied on the activation of the TGFβ-SMAD signaling pathway in cancer cells. We analyzed a set of 320 human PDAC samples by IHC, and reported that high levels of pSMAD2 (an effector downstream of TGFβ) were positively correlated with perineural invasion. More specifically, we showed by mass spectrometry that Schwann cells were a source of various secreted factors (including the TGFβ) involved in cell plasticity, migration, ECM remodeling, and potentially in the PNI process. Our results are summarized in the model presented in Fig. S9.

TGFβ is a pleiotropic cytokine implicated in wound healing, fibrosis, ECM remodeling, immune response and transformation^45,46^. TGFβ has a dual role in cancer acting either as a tumor suppressor or a tumor promoter^24,47,48^. In a previous work, we demonstrated that TGFβ pathway activation in pancreatic acinar cells facilitated PDAC initiation through its capacity to induce acinar-to-ductal cell metaplasia, providing a favorable environment for *KRAS*^*G12D*^-dependent carcinogenesis^49^. In the present study, we show that Schwann cells foster Capan-2 pancreatic cancer cell aggressiveness through TGFβ signaling, including increased migratory and invasive processes. We report that these TGFβ-induced properties of Capan-2 cells were associated with defects in epithelial and cell–cell interaction markers such as F-ACTIN, β-CATENIN and E-CADHERIN, suggesting a mechanism of epithelial-to-mesenchymal transition, known as an oncogenic process induced by TGFβ^50–52^. More generally, our results clearly demonstrate a stimulating effect of Schwann cells on PDAC cell plasticity not only to favor cancer cell migration and invasion but also to support the oncogenic properties of transformed cells such as their ability to form colonies in a harsh environment, as evidenced from survival assays and soft agar experiments. One could envision that in vivo, invaded nerves may constitute a niche for cancer cells owing to the presence of TGFβ.

Hence, in an effort to understand more precisely the functional and molecular dialog between pancreatic cancer cells and Schwann cells, we performed an original experimental approach relying on the mass spectrometry characterization of secreted factors from PDAC cells (Capan-2) and Schwann cells (sNF96.2) cultured alone or in combination. The protein signatures we identify herein match molecular networks and biological processes associated with tumor progression. More specifically, we revealed that the secretome of Schwann cells was enriched in proteins involved in biological processes related to cellular motility. This observation can be interpreted by the ability of Schwann cells to secrete factors that will potentially drive the migration of cancer cells.

We herein demonstrated that Schwann cells were a crucial driver of PDAC cancer cell aggressiveness in vitro. In our model, we used sNF96.2 Schwann cells which spontaneously express high levels of TGFβ. Previous studies reported that loss of NF1 could be responsible for increased TGFβ secretion. We then used two NF1-positive schwann cells (HSwC and ipn02.3 2λ). We observed that these two cell lines (co cultures or CM experiments) could induce the migration of cancer cells through a TGFβ-dependent mechanism, and, that they were a major source of TGFβ. These observations rule out the possibility that increased TGFβ secretion that might occur in sNF96.2 cells result from the loss of NF1. How relevant these two cell models are to Schwann cells in the in vivo PDAC microenvironment remains to be evaluated. In an attempt to address this issue, we analyzed a set of 320 human PDAC samples by IHC, and reported that high levels of pSMAD2 were positively correlated with perineural invasion. This observation strengthened a causal role for TGFβ in perineural invasion. However, we were unable to discrimite the exact member of the TGFβ superfamily involed in vivo in SMAD signaling activation, since activin and GDNFs are also known to signal through SMAD2. Finally, wether or not Schwann cells are a significant source of TGFβ in human PDAC tumors remains an open question.

Interestingly, the secretome of co-cultured Schwann cells/cancer cells, beyond its higher content in motility proteins, was also enriched in ECM proteins and adhesion molecules. Hence, it is interesting to note that these proteins are found in the stromal compartment in corroboration with previous studies. For instance, we identified proteins such as FN1, SPARC, VCAN, or WNT5A which were previously reported of poor prognosis in a PDAC stromal signature following microarray analyses on virtual microdissections^53^. In addition, SPARC^54^, SPOCK1^55^ and FN1^56^ proteins, are known to be positively correlated with PDAC invasion and progression. Our screening revealed various collagens, a large family of ECM deposition proteins known to be aberrantly expressed in cancer, activated by TGFβ^47^ and associated with metastasis and poor-prognosis in ovarian^57^and pancreatic cancer progression^58^. Moreover, our STRING analysis revealed that TGFβ1 could strongly interact with MMP2, SERPIN1, FN1 et ACTN1 proteins. MMPs are matrix metalloproteinases that are involved in carcinogenesis and positively regulated by TGFβ^59^. SERPIN1, also known as PAI-1, is the archetype of the SMAD-induced TGFβ target genes and widely described as a marker of poor prognosis, positively involved in acquisition of aggressive properties in many tumors, including PDAC^60^. FN1, a transcriptional target of TGFβ^61^ abundantly found in PDAC stroma, is a matrix glycoprotein that supports metastatic spread and chemoresistance^62^. Finally, ACTN1 expression can be induced by TGFβ^63^ and was shown to impair E-cadherin-dependent celluar adhesion and is associated with poor prognosis in breast cancer^64^. The proteomic analysis identified several potential factors that might be important in the crosstalk between cancer cells and Schwann cells, such as PDGFs (platelet-derived growth factor), IGFBPs (insulin-like growth factor-binding proteins) and CCL/CXCRs (chemokine receptors). These factors are largely documented for their role in cancer. It will be of particular interest to investigate in future studies their possible involvment in perineural invasion, as well as with their functional interaction with TGFβ signaling.

In conclusion, our work demonstrates that Schwann cells, surrounding nerve fibers during PANR, have the capacity to foster cancer cell migration and survival through different factors including TGFβ signaling. They may represent a protective and nurturing environment in vivo, promoting local relapse or distant metastases. This study paves the way for targeting new factors involved in PANR, the inhibition of which may be beneficial for improving patient quality of life and survival.

## Supplementary information

FigS1

FigS2

FigS3

FigS4

FigS5

FigS6

FigS7

FigS8

FigS9

Supplementary data 1

Supplementary data 2

Supplementary data 3

Supplementary data 4

Supplementary figures caption
