# Correction: Schwann cells support oncogenic potential of pancreatic cancer cells through TGFβ signaling

**DOI:** 10.1038/s41419-020-2262-1

**Published:** 2020-01-23

**Authors:** Elodie Roger, Sylvie Martel, Adrien Bertrand-Chapel, Arnaud Depollier, Nicolas Chuvin, Roxane M. Pommier, Karam Yacoub, Cassandre Caligaris, Victoire Cardot-Ruffino, Véronique Chauvet, Sophie Aires, Kayvan Mohkam, Jean-Yves Mabrut, Mustapha Adham, Tanguy Fenouil, Valérie Hervieu, Laura Broutier, Marie Castets, Cindy Neuzillet, Philippe A. Cassier, Richard Tomasini, Stéphanie Sentis, Laurent Bartholin

**Affiliations:** 1 Université de Lyon, Université Claude Bernard Lyon 1, INSERM 1052, CNRS 5286, Centre Léon Bérard, Centre de recherche en cancérologie de Lyon (CRCL), Lyon, 69373 France; 2 Hospices Civils de Lyon, Croix Rousse hospital, Claude-Bernard Lyon 1 University, Department of General Surgery & Liver Transplantation, Lyon, France; 3 Hospices Civils de Lyon, Edouard Herriot hospital, Claude-Bernard Lyon 1 University, Department of General Surgery & Liver Transplantation, Lyon, France; 4 Hospices Civils de Lyon Institute of Pathology EST, CRCL INSERM U1052, University Lyon 1, Lyon, France; 5 Medical Oncology Department, Curie Institute, Versailles Saint-Quentin University, 35 rue Dailly, 92210 Saint Cloud, France; 6 Departement d’Oncologie Médicale, Centre Léon Bérard, Lyon, 69008 France; 7 Aix-Marseille Université, Institut Paoli-Calmettes, INSERM U1068, CNRS UMR 7258, Centre de Recherche en Cancérologie de Marseille, Marseille, France; 8 Present Address: Clinical Research Division, Fred Hutchinson Cancer Research Center, Seattle, WA USA

**Keywords:** Cancer microenvironment, Oncogenes

**Correction to**: **Cell Death and Disease**

10.1038/s41419-019-2116-x published online 25 November 2019

The original version of this article contained an error in the name of one of the co-authors (Kayvan Mohkam). This has been corrected in the PDF and HTML versions.

