# Supplementary figures and images for: Schwann cells support oncogenic potential of pancreatic cancer cells through TGFβ signaling
Source: Cell Death Dis. 2019 Nov 25;10(12):886. doi: 10.1038/s41419-019-2116-x (PMC6877617; doi:10.1038/s41419-019-2116-x)

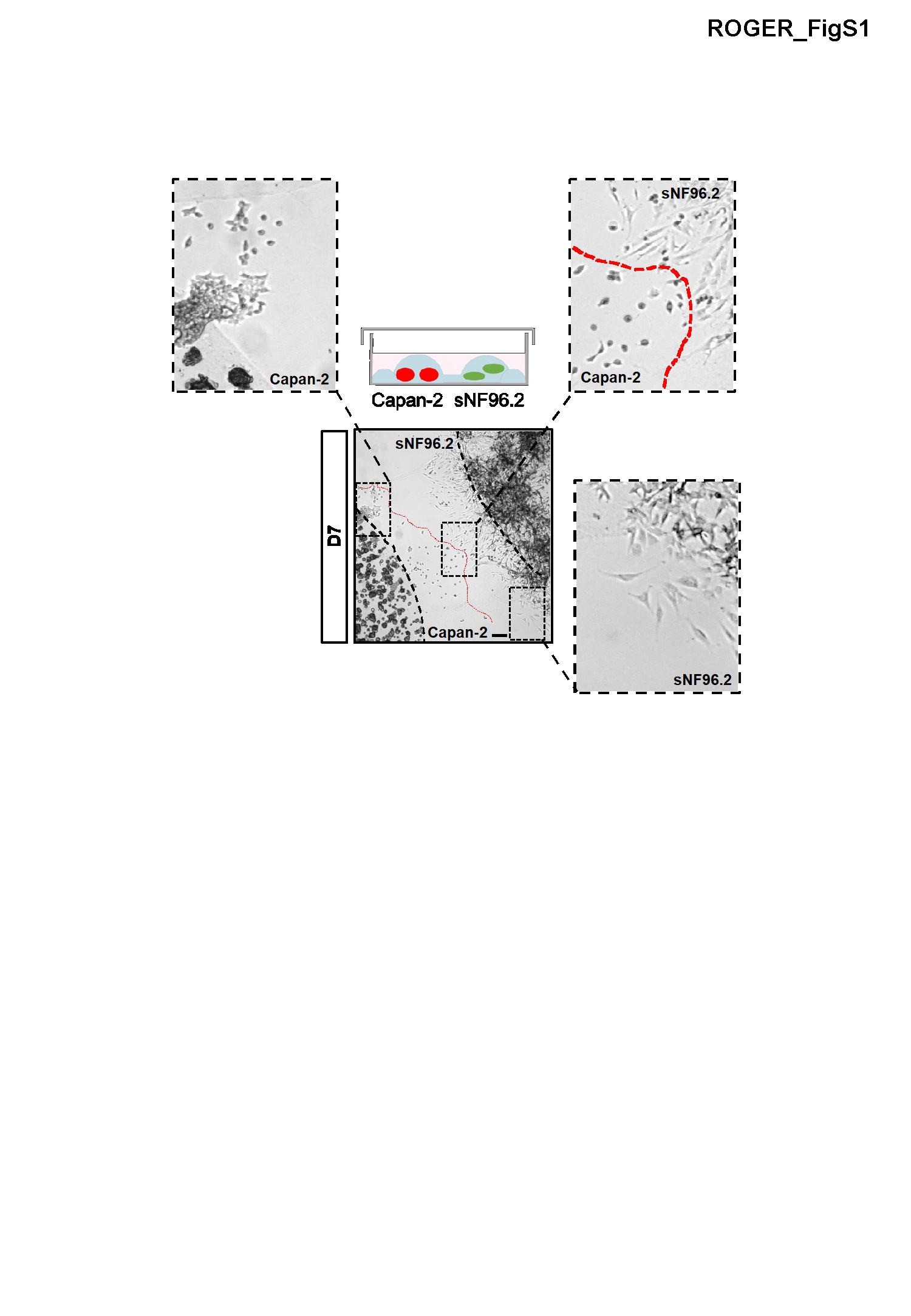

Supplement: Supplementary file 1 — FigS1 [file 41419_2019_2116_MOESM1_ESM.tif]

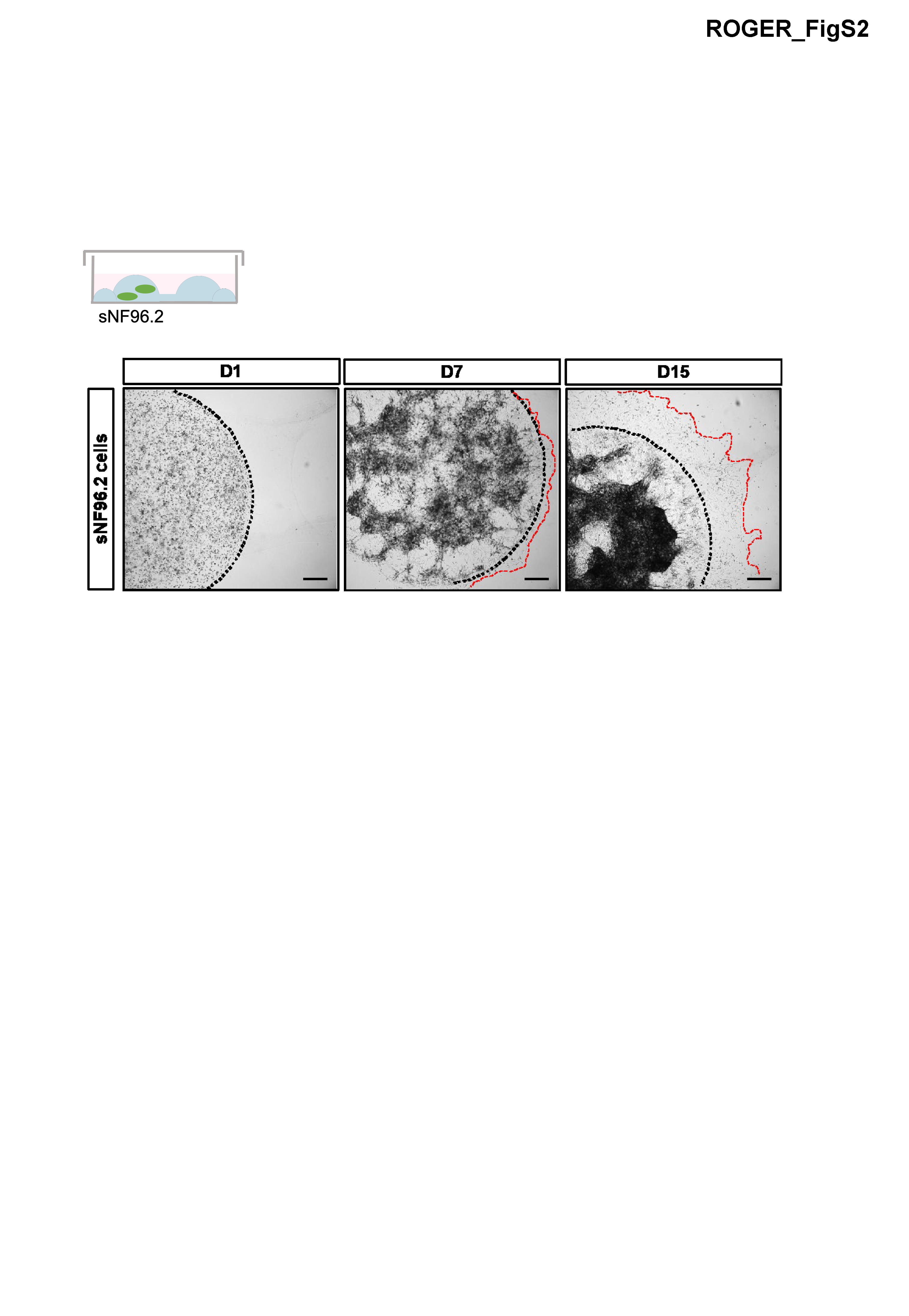

Supplement: Supplementary file 2 — FigS2 [file 41419_2019_2116_MOESM2_ESM.tif]

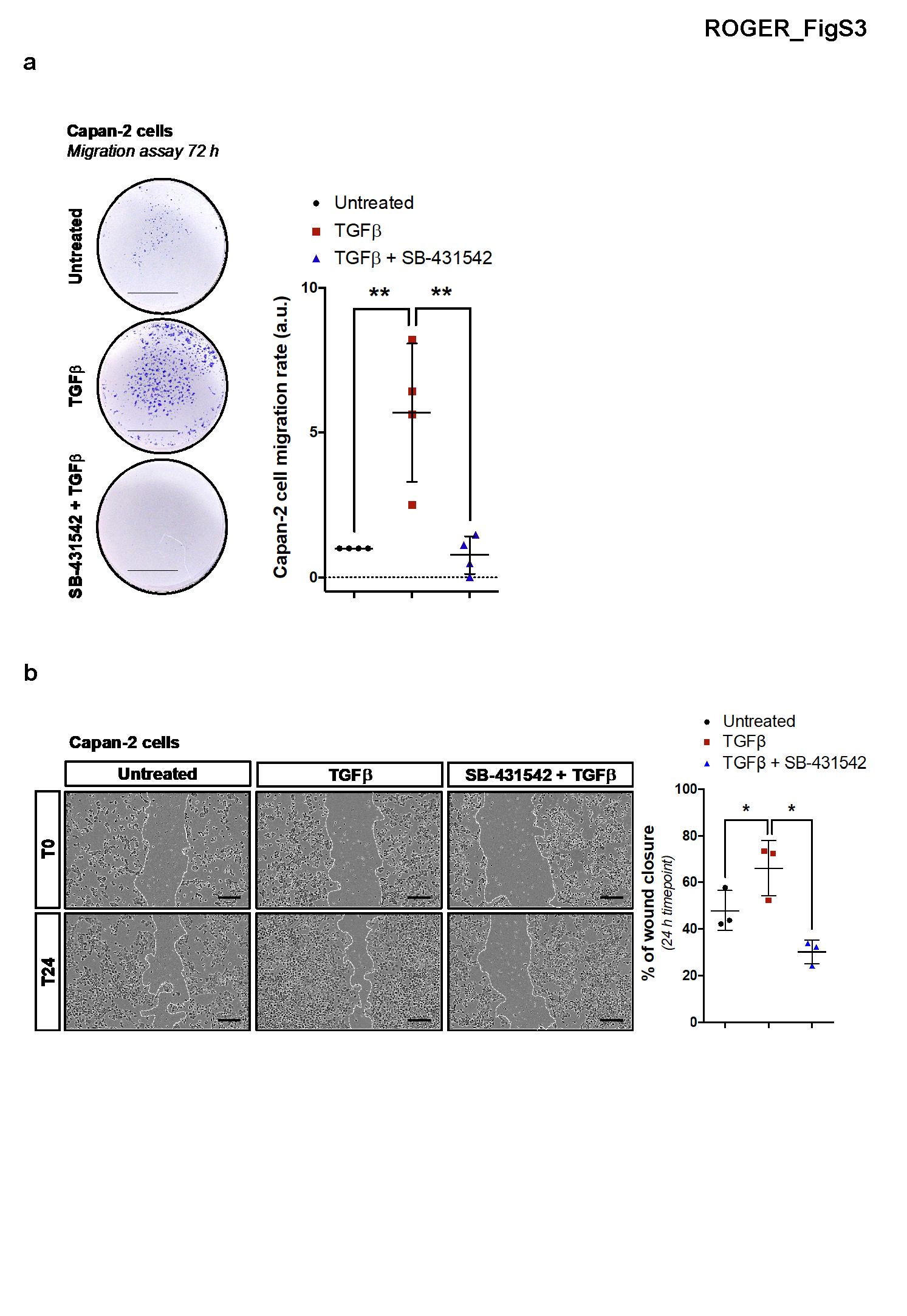

Supplement: Supplementary file 3 — FigS3 [file 41419_2019_2116_MOESM3_ESM.tif]

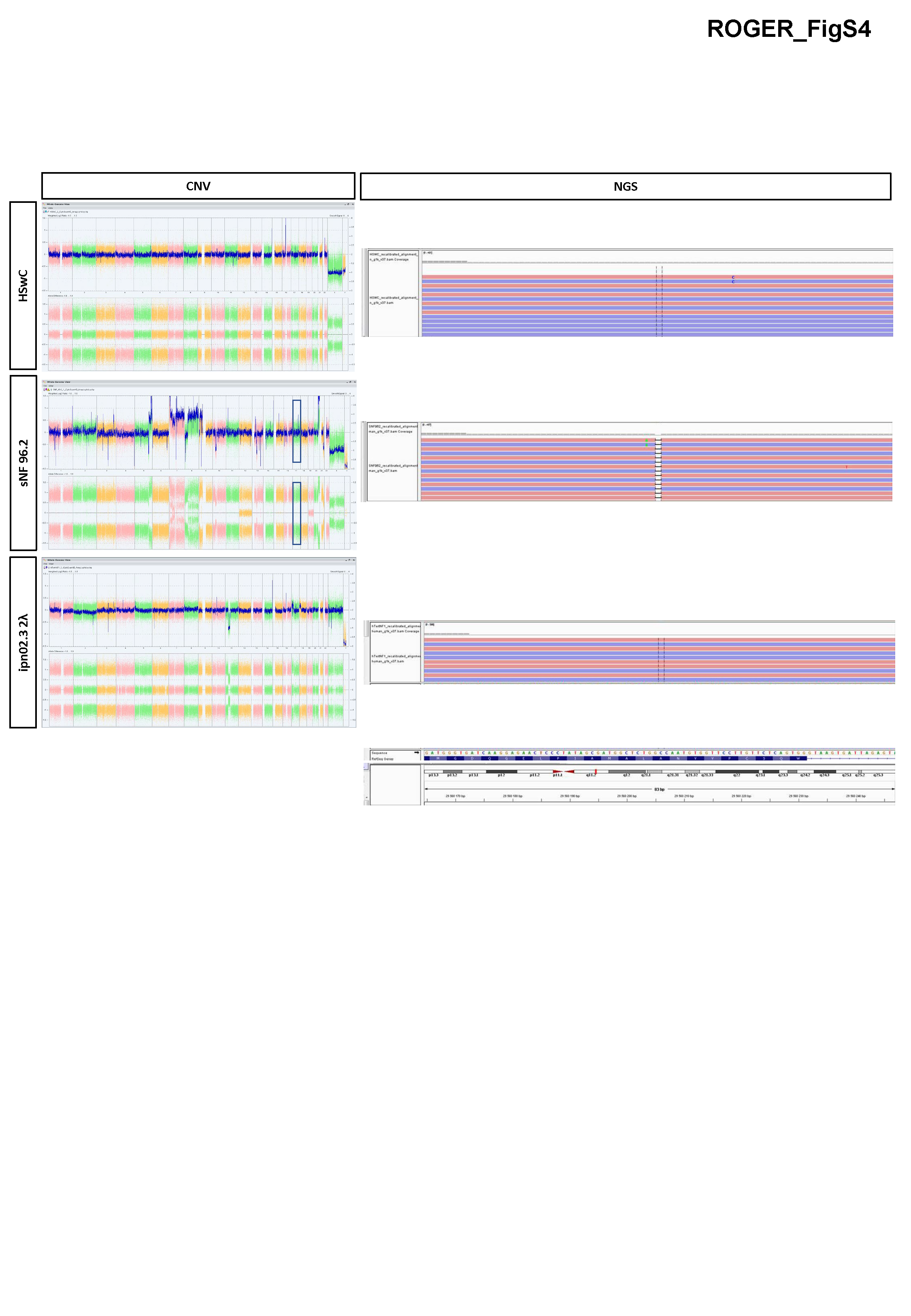

Supplement: Supplementary file 4 — FigS4 [file 41419_2019_2116_MOESM4_ESM.tif]

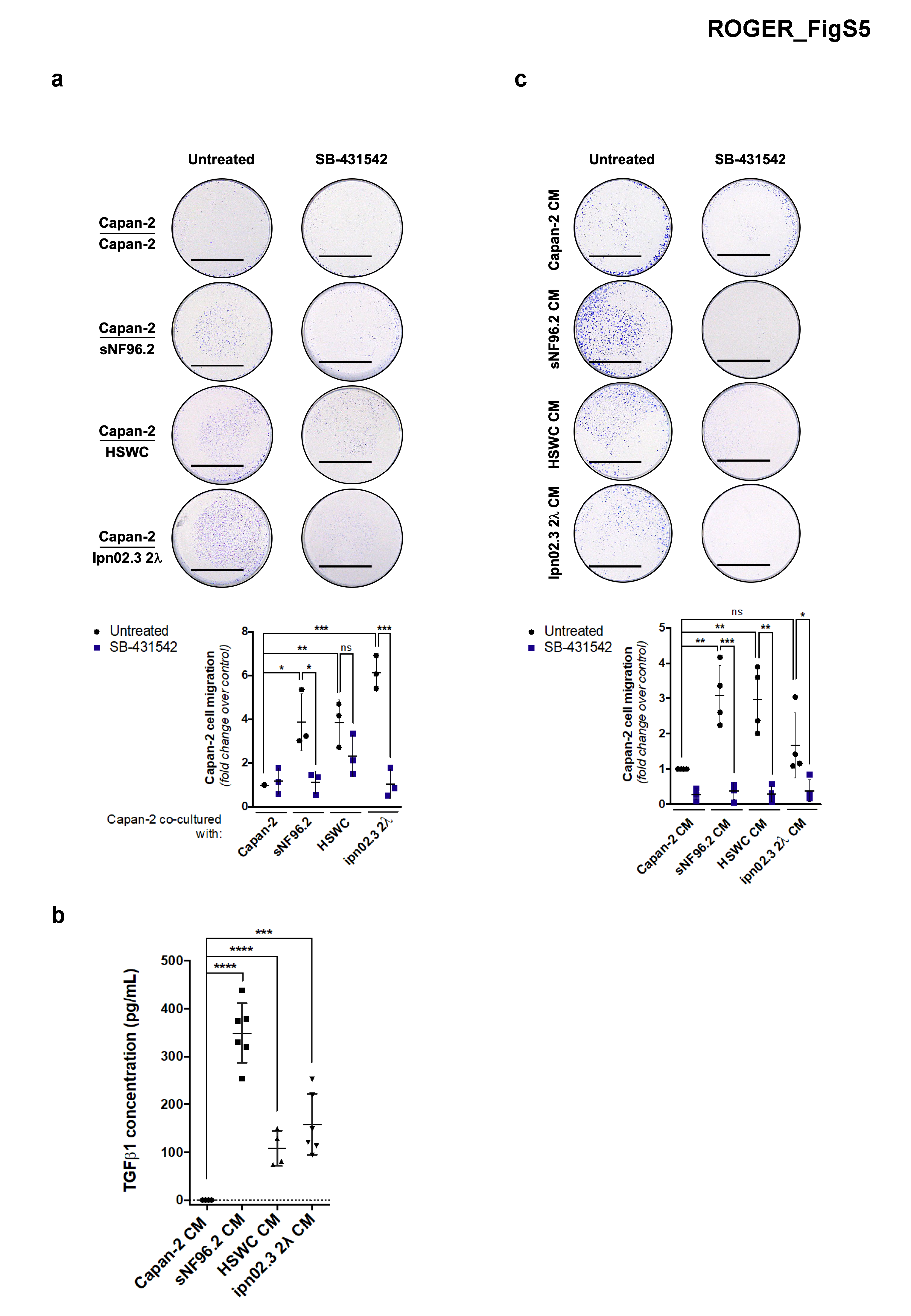

Supplement: Supplementary file 5 — FigS5 [file 41419_2019_2116_MOESM5_ESM.tif]

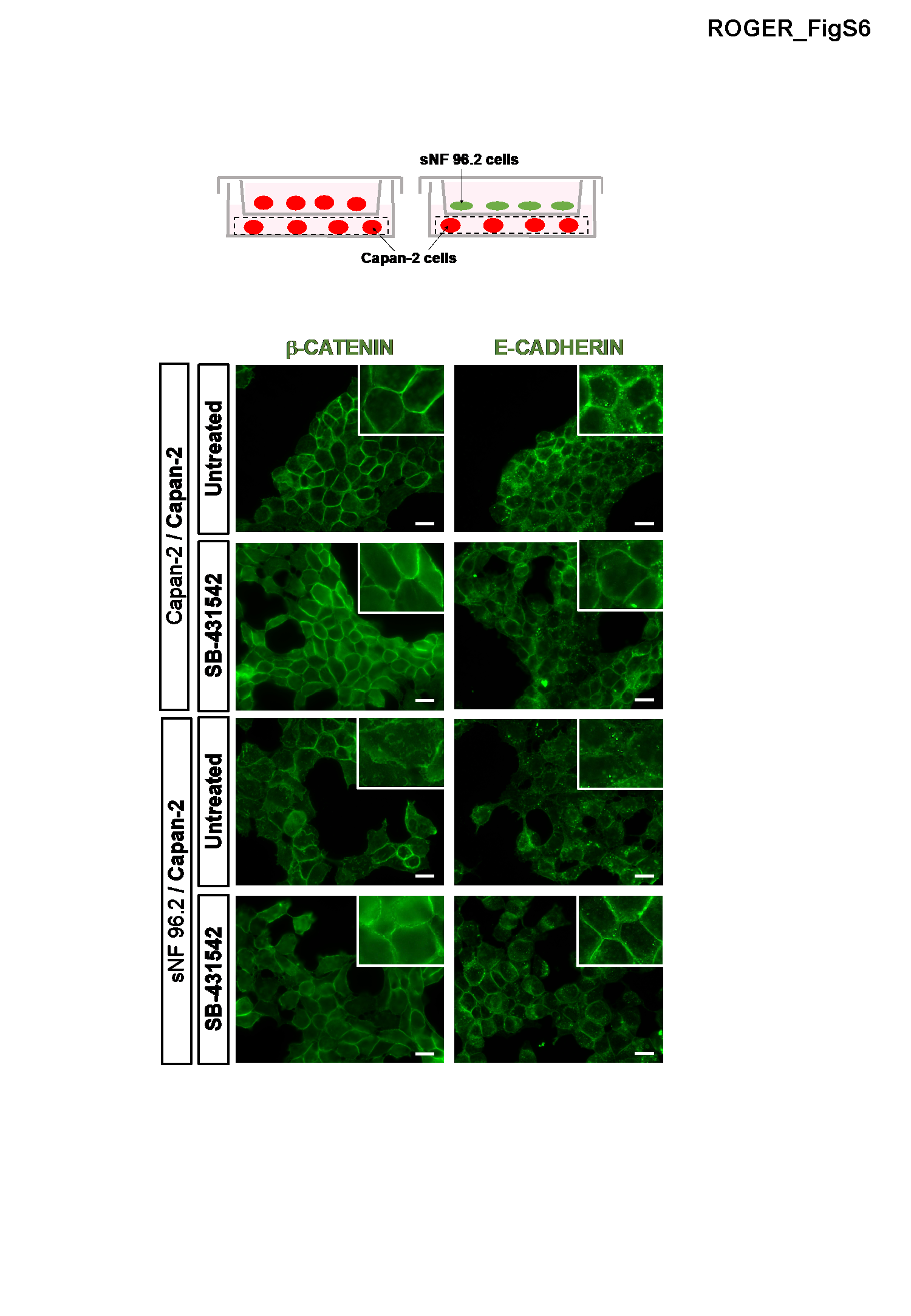

Supplement: Supplementary file 6 — FigS6 [file 41419_2019_2116_MOESM6_ESM.tif]

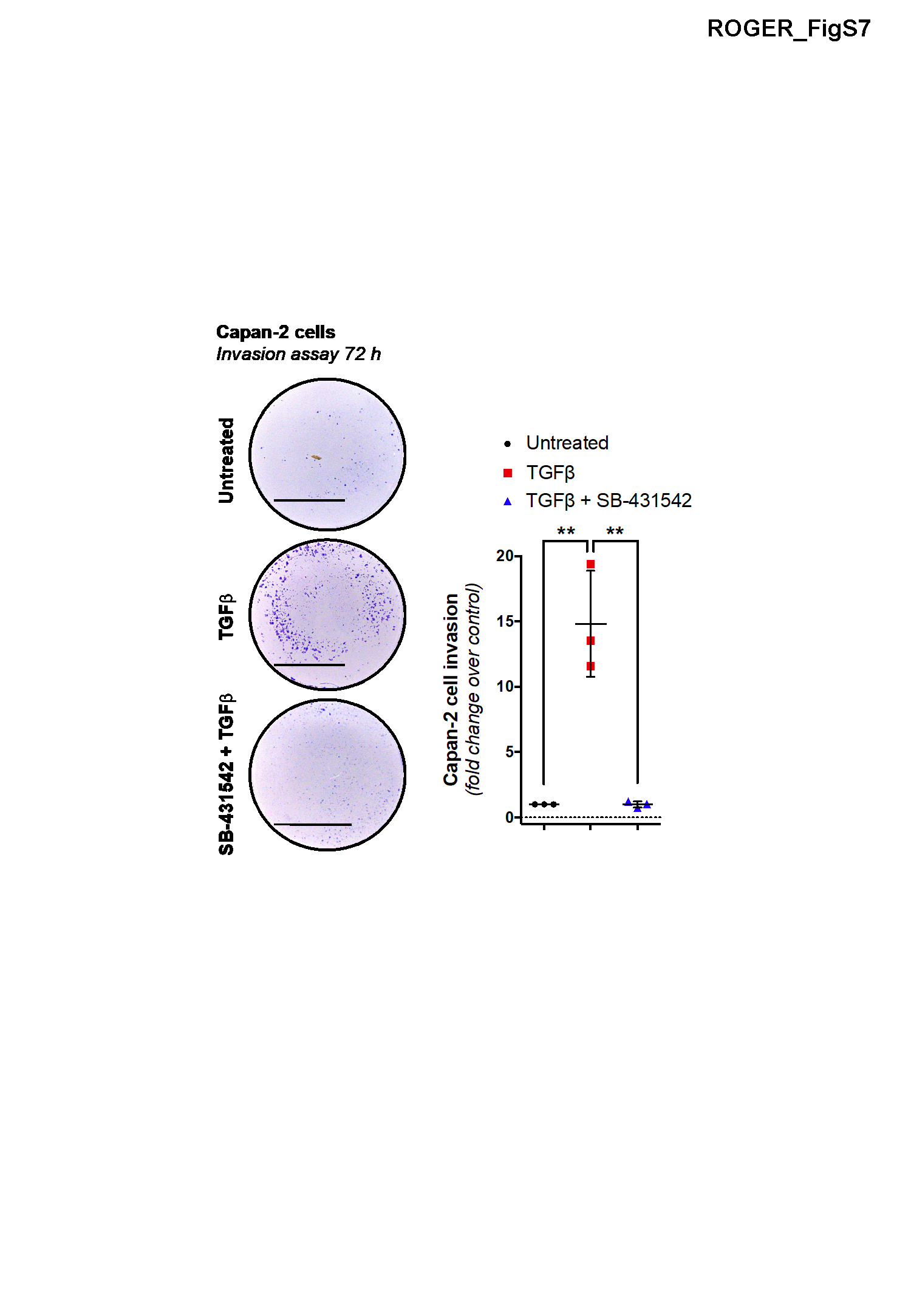

Supplement: Supplementary file 7 — FigS7 [file 41419_2019_2116_MOESM7_ESM.tif]

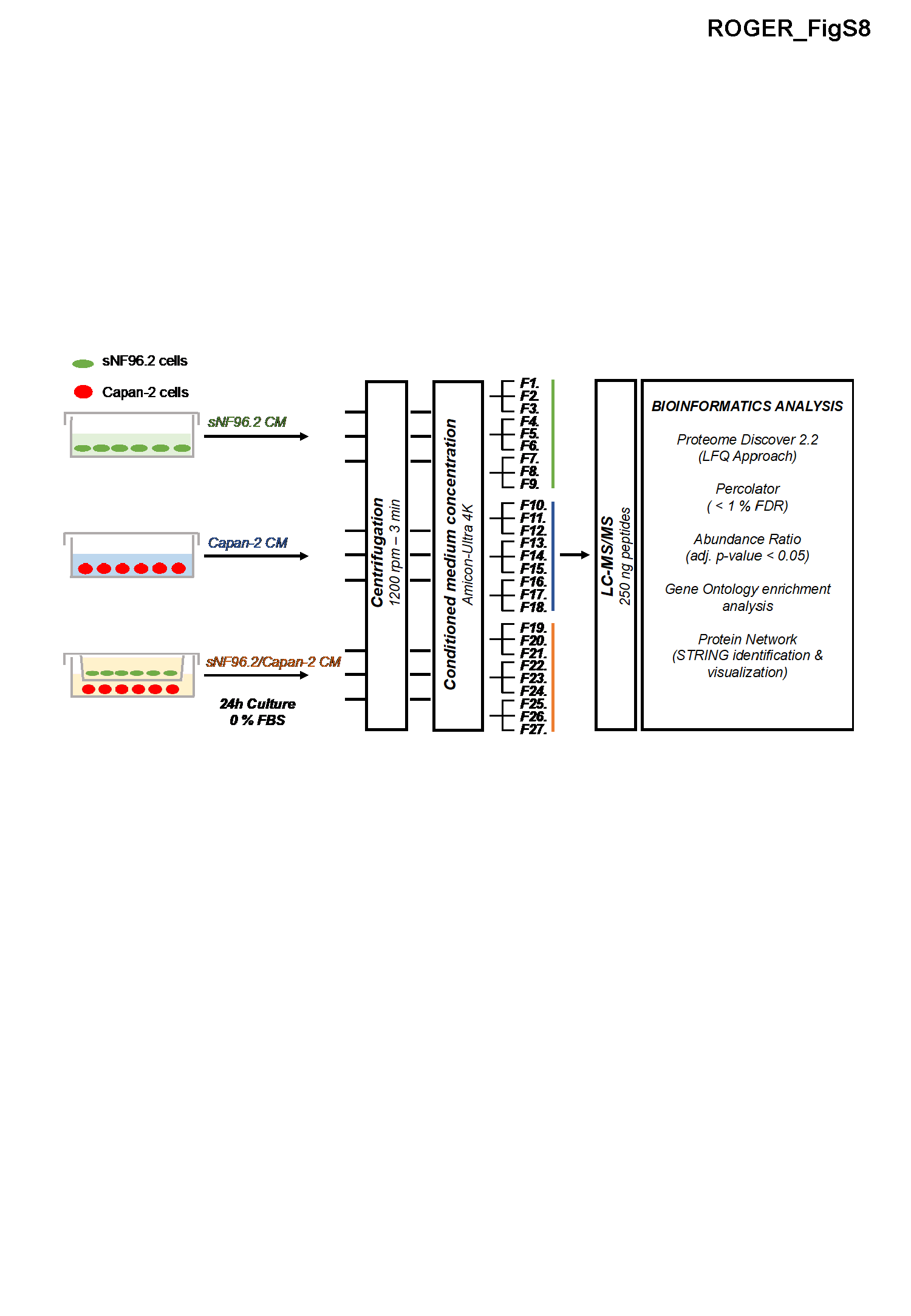

Supplement: Supplementary file 8 — FigS8 [file 41419_2019_2116_MOESM8_ESM.tif]

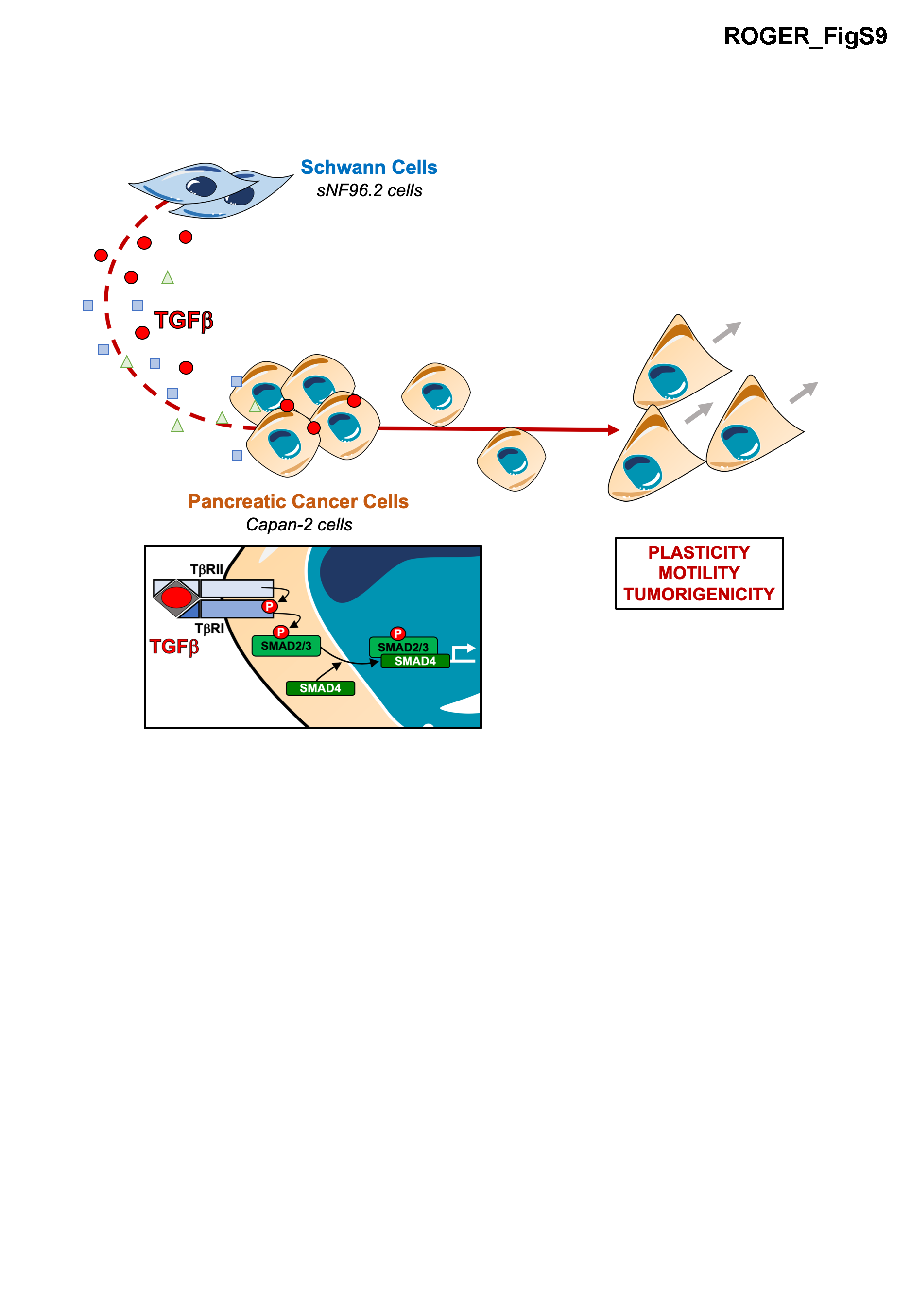

Supplement: Supplementary file 9 — FigS9 [file 41419_2019_2116_MOESM9_ESM.tif]
