# Supplementary material for: Schwann cells support oncogenic potential of pancreatic cancer cells through TGFβ signaling
Source: Cell Death Dis. 2019 Nov 25;10(12):886. doi: 10.1038/s41419-019-2116-x (PMC6877617; doi:10.1038/s41419-019-2116-x)
Supplement: Supplementary file 14 — Supplementary figures caption [file 41419_2019_2116_MOESM14_ESM.docx]

## Figure S1. Cell shape of Capan-2 cells and sNF96.2 cells within three-dimensional motility assay

Three-dimensional motility assay of Capan-2 cells co-cultured with sNF96.2 cells in matrigel drops at day 7 (D7). The black dotted lines indicate the cell localization at the beginning of the experiment. The red dotted lines represent the Capan-2 cell migration front. Representative areas of Capan-2 cells and sNF96.2 cells (black frames) are enlarged. Bright field images are representative of one experiment performed three times. Scale bar, 200 μm.

## Figure S2. sNF96.2 cells display motility capabilities independently of Capan-2 cells

Three-dimensional motility assay of sNF96.2 cells cultured in matrigel drops for 15 days. The black dotted lines indicate the cell localization at the beginning of the experiment. The red dotted lines represent the sNF96.2 cell migration front. Bright field images at days 1 (D1), 7 (D7) and 15 (D15) are representative of one experiment performed three times. Scale bars, 500 μm.

## Figure S3. TGFβ-enhanced Capan-2 cell migration is suppressed by the TGFβ type I-receptor inhibitor, SB-431542

**a** Boyden chamber migration assay of Capan-2 cells cultured for 72 h, treated or not with TGFβ and SB-431542 TβRI inhibitor and stained with a 0.1% crystal violet solution. For each condition, an image from one experiment representative of four independent experiments is shown (left panel) and Capan-2 cell migration quantification is represented as mean ± SD (right panel, n = 4 independent experiments, ** *P* < 0.01). Scale bars, 0.5 cm. **b** Wound healing assay of Capan-2 cells cultured for 24 h, treated or not with TGFβ and SB-431542. Bright field images at h 0 (T0), and 24 (T24) are representative of one experiment performed three times (left panel) and quantification of wound closure is represented as mean ± SD (right panel, n = 3 independent experiments, significance is shown for T24 conditions, **P* < 0.05; ** *P* < 0.01). Scale bars, 400 μm. CM, conditioned medium.

## Figure S4. Genetic analyses of the sNF96.2, ipn02.3 2λ and HSwC cells to determine their NF1 status

Left panel: Comparison of copy number profiles of NF96.2, ipn02.3 2λ and HSwC Schwann cells. The sNF96.2 cell line harbors a chr17 copy neutral LOH (within the rectangle). Right panel: IGV sequence comparison of three cell lines. The sNF96.2 cell line harbors a NF1 frameshift mutation (c.3683delC; p.A1228fs) with a 100 % variant allele frequency. IGV, integrative genomics viewer; LOH, loss of heterozygity; CNV, copy-number variation; NGS, next generation sequencing

## Figure S5. sNF96.2, ipn02.3 2λ and HSwC Schwann cells secrete high amounts of TGFβ and increase the TGFβ-dependent motility of Capan-2 pancreatic cancer cells

**a** Boyden chamber migration assay of Capan-2 cells cultured for 72 h alone (Capan-2 / Capan-2 condition) or with indicated Schwann cells (Capan-2 / sNF96.2; Capan-2 / ipn02.3 2λ; Capan-2 / HSwC conditions), treated or not with SB-431542, and stained with a 0.1% Crystal violet solution. For each condition, an image from one experiment representative of three independent experiments is shown (top panel) and Capan-2 cells migration quantification is represented as mean ± SD (bottom panel, n = 3 independent experiments, * *P* < 0.05; ** *P* < 0.01; *** *P* < 0.001; ns: not significant). Scale bars, 0.5 cm**. b** Amount of soluble TGFβ1 secreted into the medium by Capan-2 cells (Capan-2 CM) and by indicated Schwann cells (sNF96.2 CM, HSwC CM and ipn02.3 2λ CM). Quantification of soluble TGFβ1 concentration is represented as mean ± SD (n = at least 4 independent biological replicates, *** *P* < 0.001, **** *P* < 0.0001). **c** Boyden chamber migration assay of Capan-2 cells cultured for 72 h either with Capan-2 CM or with sNF96.2 CM or with ipn02.3 2λ CM or with HSwC CM, treated or not with the TβRI inhibitor, SB-431542, and stained with a 0.1% crystal violet solution. For each condition, an image from one experiment representative of four independent experiments is shown (top panel) and Capan-2 cell migration quantification is represented as mean ± SD (bottom panel, n = 4 independent experiments, * *P* < 0.05; ** *P* < 0.01; *** *P* < 0.001; ns: not significant). Scale bars, 0.5 cm.

## Figure S6. sNF96.2 cells modulate the Capan-2 cell-cell cohesion through TGFβ signaling

Immunofluorescence detection of β-CATENIN and E-CADHERIN proteins in Capan-2 cells co-cultured either with Capan-2 cells (Capan-2 / Capan-2 condition) or with sNF96.2 cells (sNF96.2 / Capan-2 condition) and treated or not with SB-431542 TβRI inhibitor for 24 h. Scale bars, 15 μm.

## Figure S7. TGFβ-enhanced Capan-2 cell invasion is suppressed by the TGFβ type I-receptor inhibitor, SB-431542

Boyden chamber invasion assay of Capan-2 cells cultured in matrigel for 72 h, treated or not with TGFβ and SB-431542 TβRI inhibitor and stained with a 0.1% Crystal violet solution. For each condition, an image from one experiment representative of three independent experiments is shown (left panel) and Capan-2 cell migration quantification is represented as mean ± SD (right panel, n = 3 independent experiments, ** *P* < 0.01). Scale bars, 0.5 cm.

## Figure S8. Proteomic analysis of conditioned medium from Capan-2 and sNF96.2 cells cultured alone or in combination

Workflow of the LFQ mass spectrometry experiment. Nine technical replications of sNF96.2 CM, Capan-2 CM and sNF96.2/Capan-2 CM were used for proteomic analyses; the resulting peptides were analyzed by LC-MS/MS and quantified with the label-free algorithm in Proteome Discover 2.2 software. The significant proteins were analyzed by gene ontology and protein-protein interaction network analyses.

## Figure S9. Model of Capan-2 cell oncogenic potential supported by sNF96.2 cells

sNF96.2 cell, confronted with Capan-2 cells, exhibit the ability to secrete several factors involved in the motility Capan-2 cells. The TGFβ cytokine is identified as one of these factors, contributing to the establishment of an effective dialogue between Capan-2 cells and sNF96.2 cells. Activation of the TGFβ-SMAD signaling pathway within Capan-2 cells, in response to sNF96.2 cells, is correlated with the modulation of their cell-cell junctional complex, with an increase in their spreading behavior and finally with their oncogenic potential.
